# Snakes of the Pernambuco Endemism Center, Brazil: diversity, natural history and conservation

**DOI:** 10.3897/zookeys.1002.50997

**Published:** 2020-12-10

**Authors:** Rafaela C. França, Mayara Morais, Frederico G. R. França, Dennis Rödder, Mirco Solé

**Affiliations:** 1 Programa de Pós-graduação em Ecologia e Conservação da Biodiversidade, Universidade Estadual de Santa Cruz, Rodovia Jorge Amado, Km 16, CEP 45662-900 Ilhéus, Bahia, Brazil; 2 Herpetology Section, Zoologisches Forschungsmuseum Alexander Koenig, Adenauerallee 160, 53113 Bonn, Germany; 3 Programa de Pós-graduação em Zoologia, Universidade Estadual de Santa Cruz, 11 Rodovia Jorge Amado, Km 16, CEP 45662-900 Ilhéus, Bahia, Brazil; 4 Departamento de Engenharia e Meio Ambiente, Centro de Ciências Aplicadas e Educação, Universidade Federal da Paraíba – UFPB, Av. Santa Elizabete, s/n – Centro. CEP 58297-000, Rio Tinto, PB, Brazil; 5 Departamento de Ciências Biológicas, Universidade Estadual de Santa Cruz, Rodovia Jorge Amado, km,16, 45662-900 Ilhéus, Bahia, Brazil

**Keywords:** biodiversity, inventory, geographic distribution, natural history, Serpentes, richness

## Abstract

The Atlantic Forest is one of the largest and richest tropical rainforests on the planet, being one of the 25 world priorities for conservation. The Atlantic Forest portion located north of the São Francisco River corresponds to the Pernambuco Endemism Center (PEC). We describe the snake composition of the PEC, providing information about the diversity, natural history and geographical distribution of the species, based on records from five scientific collections and additional information from the literature. A total of 78 species of snakes distributed in eight families was registered in the Pernambuco Endemism Center. The Caatinga is the Brazilian biome that most shares species with the PEC, followed by Cerrado. On the other hand, seven species are considered endemic of this region. Most of the snake species in the PEC have been registered in forest (94.8%), followed by “Brejos Nordestinos” (46.1%), Tabuleiros (43.5%), Restingas (14.1%) and Mangroves (5.1%). The PEC snake fauna includes mainly terrestrial species (60.2%) and cryptozoic and/or fossorial species (21.7%), but also presents a high richness of semi-arboreal and arboreal species (29.5%). Vertebrates are the main food item consumed by the species (78% of species), among the main prey are mammals, lizards, and amphibians. Most species show a strictly nocturnal activity period (50%), followed by strictly diurnal (38%). The PEC is the most degraded and least known region of the Atlantic Forest, yet it has revealed a high richness of snake species, including seven endemic species. It is emphasized that regional conservation efforts need to be intensified, because few forests in the region are formally protected, and the majority consist of small and poorly protected fragments, which means that many species in the region may be in risk of extinction.

## Introduction

The Atlantic Forest is considered one of the 25 priority areas for conservation worldwide (Myers et al. 2000). This biome was one of the largest tropical forests in the Americas, originally covering 150 million hectares along the Brazilian coast and parts of Paraguay and Argentina (Silva and Casteleti 2003). Today, the Atlantic Forest has been reduced to less than 12% of its original coverage (Ribeiro et al. 2009). Even having suffered an extensive fragmentation since long time ago, the Atlantic Forest still presents a great biodiversity, housing one of the highest percentages of endemic species in the world (Morellato and Haddad 2000).

Although practically the entire Brazilian coast was occupied by European colonization, it was in the northeast that the Atlantic Forest was more rapidly degraded, due to the economic cycle of brazilwood and sugar cane (Coimbra-Filho and Câmara 1996). This degradation is even more evident in the portion of the Atlantic Forest located north of the São Francisco River, where an important center of endemism is located in South America – The Pernambuco Endemism Center (hereafter PEC) (Prance 1982, Silva and Casteleti 2003). In this region, sugar cane is the main agricultural crop and other anthropic actions, such as animal and plant extractivism, have contributed to the reduction of biodiversity in the PEC (Coimbra-Filho and Câmara 1996, Tabarelli et al. 2002, 2006a). In the midst of this scenario, the PEC is considered the most devastated, least known and least protected sector of the Atlantic Forest, being one of the regions on the planet where conservation efforts are most urgent (Coimbra-Filho and Câmara 1996, Tabarelli et al. 2002, 2005).

Among reptiles, snakes are the group that currently presents the most underestimated risks of extinction, due to the scarcity of information on the natural history of most species, mainly because they have long periods of inactivity, are difficult to observe and live in low population densities (Seigel 1993). Although some studies carried out on Atlantic forest remnants of the PEC have provided important information about snakes in this region (e.g. Moura et al. 2011, Pereira Filho and Montingelli 2011, França et al. 2012, Roberto et al. 2012, 2015, Rodrigues et al. 2015, Pereira Filho et al. 2017, Mesquita et al. 2018, Sampaio et al. 2018, Freitas et al. 2019a), the knowledge about the diversity, distribution and natural history of PEC snake species remains scarce and fragmented. In this direction, scientific collections perform a fundamental role in obtaining information that is the basis for the description of new species, biodiversity inventories and identification of endemism areas (Rocha et al. 2014).

Herein, we describe the snake composition at the Pernambuco Endemism Center, providing information about the diversity, natural history and geographical distribution of the species, based on records from scientific collections and additional information from the literature.

## Materials and methods

### Study area

**Figure 1.**
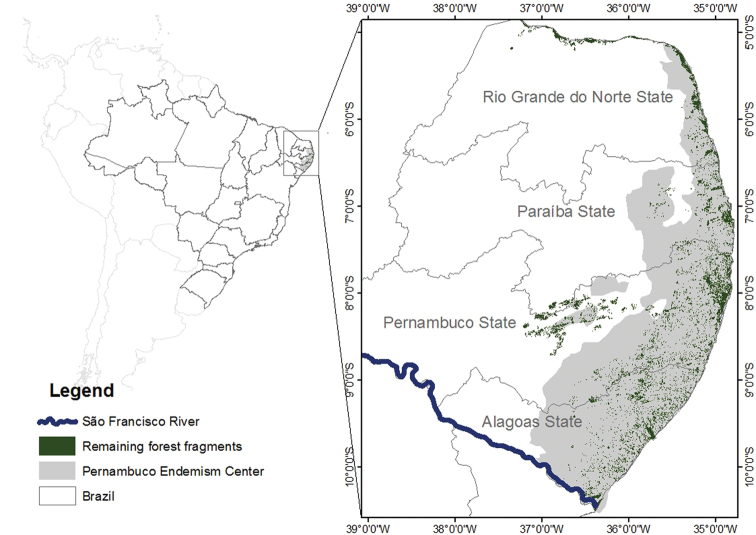
Map of the location of the Pernambuco Endemism Center, with the original coverage of Atlantic Forest (gray), and the actual remnants (green).

The study area comprises the Atlantic Forest located north of the São Francisco River, which corresponds to the Pernambuco Endemism Center (PEC) (Fig. 1) (Prance 1982, Silva and Casteleti 2003), located between the states of Alagoas and Rio Grande do Norte. This region has a humid tropical climate (Köppen’s As’), with autumn-winter rains and rainfall ranging from 750 to 1500 mm per year (Tabarelli et al. 2006a).

The PEC region is composed by different native forest formations and ecosystems associated with the Atlantic Forest domain. A mosaic of ombrophilous and semi-deciduous forests is present in this region (Tabarelli et al. 2006a). Also, PEC comprises the “Brejos de Altitude” or “Brejos Nordestinos”, which are “islands” of humid forests established in the semi-arid region, surrounded by Caatinga vegetation (Andrade-Lima 1982). Although the vegetation of the PEC is composed mainly of humid tropical forests, we can also find open physiognomies along the coast, which are called “Restingas”, and in the interior, which are called “Tabuleiros”. The restingas are formed by strips of beaches and dunes covered by herbaceous and shrubby vegetation (Araujo 1992). The Tabuleiros are considered natural enclaves of savannah, characterized by herbaceous vegetation, with scattered trees and shrubs or grouped in patches that are structurally similar to the coastal restingas, but without the marine influence (Andrade-Lima 1982). On the coast along the PEC, we can also find areas of mangroves, with a diversified aggregation of trees and shrubs that form the dominant plant communities in saline solution of the tides (Tabarelli et al. 2006b).

According to Uchoa Neto and Tabarelli (2002), the PEC presents the largest amount of remaining area of Atlantic Forest in the state of Pernambuco (1,363.23 km²), followed by the states of Alagoas (807.95 km²), Rio Grande do Norte (567.67 km²) and Paraíba (566.09 km²).

### Data collection

The data presented here is the result of verification of 3,118 snake specimens deposited in five scientific collections (Coleção Herpetológica da Universidade Federal da Paraíba – UFPB; Coleção do Laboratório de Anfíbios e Répteis da Universidade Federal do Rio Grande do Norte - CLAR; Coleção Herpetológica do Museu de História Natural da Universidade Federal de Alagoas – MUFAL; Coleção Herpetológica da Universidade Federal Rural de Pernambuco – CHUFRPE; Coleção Herpetológica da Universidade Federal de Pernambuco – CHUFPE) and literature data.

The information on the distribution and occurrence of species in each environment were obtained through the records of the scientific collections and literature data, and was subsequently georeferenced. We include records of occurrence of species in the literature only when we were able to confirm the record by direct observation, photo or through museum records or documented vouchers. Information on diet, habitat use, and litter size of the species was obtained from personal data, records of scientific collections and literature data. We categorized the snake size considering the mean body size of each species based on published data as small (< 500mm), moderate (501–1000mm) and large (> 1001mm).

**Figure 2.**
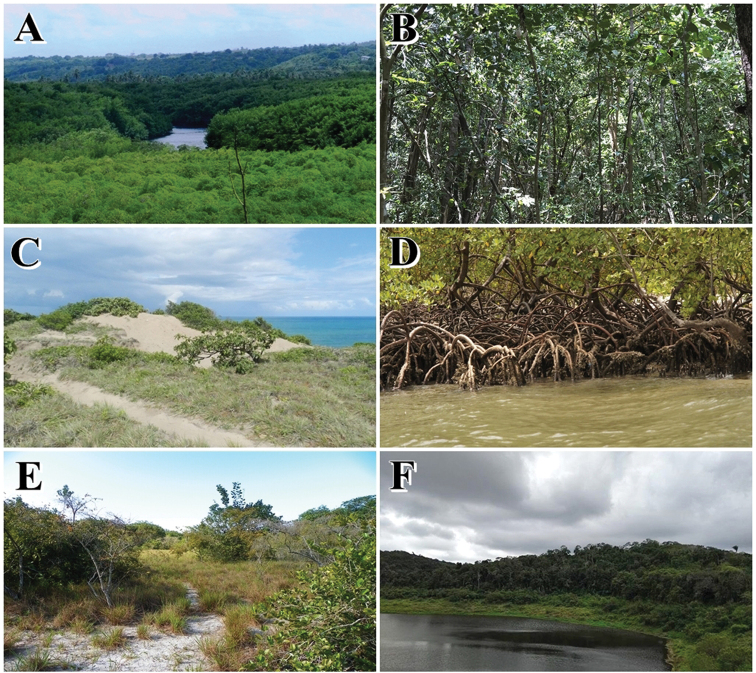
Vegetation physiognomies found in the Pernambuco Endemism Center. **A** forest **B** forest interior **C** Coastal Restingas **D** mangroves, **E** tabuleiros **F** Brejos Nordestinos. Photograph credits: Ivan L. Sampaio, in the Barra de Gramame (**A**), Frederico França, in the APA da Barra do Rio Mamanguape (**B, C**), Marcelo Melo, in the APA da Barra do Rio Mamanguape (**D**), Frederico França, in the Reserva Biológica Guaribas (**E**) and Adonias Teixeira, in the Parque Estadual Mata do Pau-Ferro (**F**).

In this work, we have differentiated the habitats of the species into five vegetation physiognomies found in this region: Forests (when the species were found in areas with a typical forest physiognomy, with a large vegetation cover, reaching 35 meters high in the canopy, presenting epiphytes, lianas and bromeliads); Coastal Restingas; Mangroves; Tabuleiros; Brejos Nordestinos (remnants of humid forests scattered in the Caatinga) (Fig. 2); and urban areas. In addition, we compared the snake fauna found in the PEC with these of five other natural ecoregions in Brazil (Amazon, Caatinga, Cerrado, Pampas, and Pantanal). These regions are divided on the basis of geomorphology, climate, and vegetation (IBGE 2004).

### Taxonomic considerations

The species *Caaeteboia* sp. found in the PEC, differs from *Caaeteboia amarali* (at present the only representative of the genus) mainly because it presents 15 rows of dorsal scales without reduction, while *C. amarali* presents 17 rows of dorsal scales without reduction. In addition, there is a strong variation between the number of ventral and subcaudal scales between the two species (Pereira Filho et al. 2017).

We decided to use the name *Micrurus ibiboboca* according to Silva Jr (2016). Although Silva Jr (2016) affirms that *M. ibiboboca* may be a species complex throughout the distribution of the species, the author still maintains the proper name. Thus, the species designated here as *M. ibiboboca* is the same mentioned in previous works as Micrurus aff. ibiboboca (e.g. França et al. 2012, Rodrigues et al. 2015, França and França 2019).

## Results

**Figure 3.**
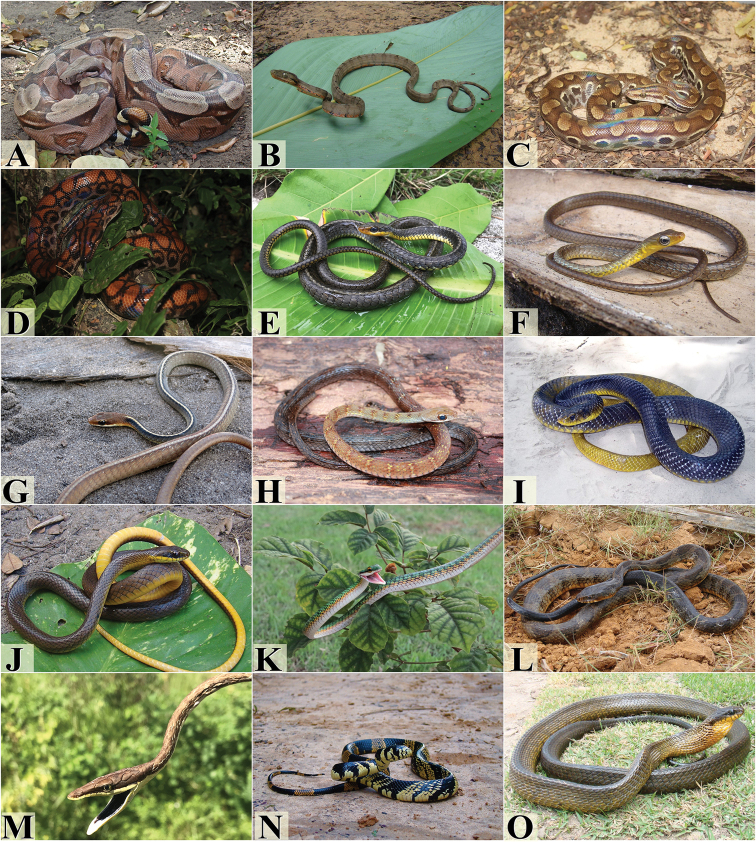
Snake species from the Pernambuco Endemism Center. **A***Boa constrictor***B***Corallus hortulanus***C***Epicrates assisi***D***Epicrates cenchria***E***Chironius carinatus***F***Chironius exoletus***G***Chironius flavolineatus*, **H***Dendrophidion atlantica***I***Drymarchon corais***J***Drymoluber dichrous***K***Leptophis ahaetulla***L***Palusophis bifossatus***M***Oxybelis aeneus***N***Spilotes pullatus***O***Spilotes sulphureus*. Photograph credits: Frederico França (**A, B, E, F, G, J, L, M, N, O**), Vanessa Nascimento (**D**), Davi Pantoja (**C, H, I**), Rafaela França (**K**).

**Figure 4.**
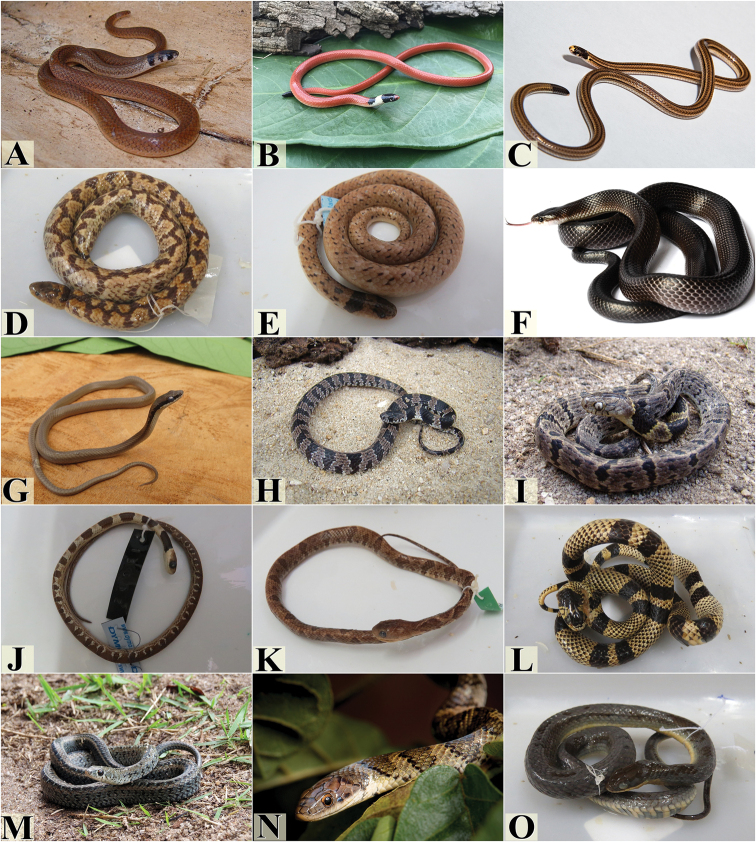
Snake species from the Pernambuco Endemism Center. **A***Tantilla melanocephala***B***Apostolepis cearensis***C***Apostolepis longicaudata***D***Atractus maculatus***E***Atractus potschi***F***Boiruna sertaneja***G***Caaeteboia* sp. **H***Dipsas mikanii***I***Dipsas neuwiedi***J***Dipsas sazimai***K***Dipsas variegata***L***Erythrolamprus aesculapii***M***Erythrolamprus almadensis***N***Erythrolamprus poecilogyrus***O***Erythrolamprus reginae*. Photograph credits: Frederico França (**A, B, G, H, I**), Anderson A. Santos (**C, N**), Rafaela França (**D, E, J, K, L, M, O**), Paulo R. S. Freitas (**F**).

**Figure 5.**
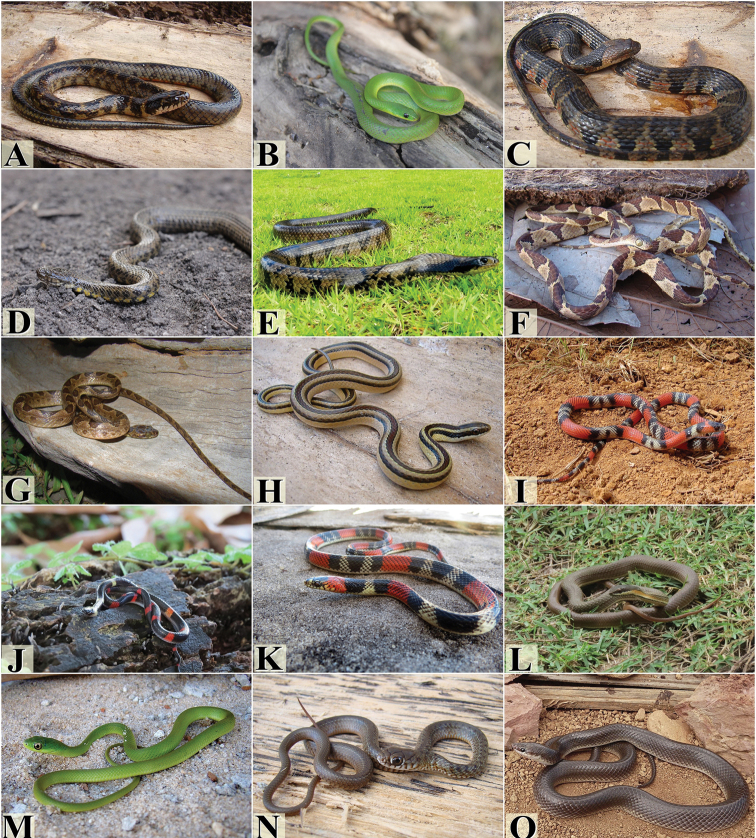
Snake species from the Pernambuco Endemism Center. **A***Erythrolamprus taeniogaster***B***Erythrolamprus viridis***C***Helicops angulatus***D***Helicops leopardinus***E***Hydrodynastes gigas***F***Imantodes cenchoa***G***Leptodeira annulata***H***Lygophis dilepis***I***Oxyrhopus guibei***J***Oxyrhopus petolarius***K***Oxyrhopus trigeminus***L***Philodryas nattereri***M***Philodryas olfersii***N***Philodryas patagoniensis***O***Phimophis guerini*. Photograph credits: Frederico França (**A, C, F, H, I, K, M, N, O**), Vanessa Nascimento (**B, D**), Ivan L. Sampaio (**E**), Willianilson Pessoa (**G**), Rafaela França (**J, L**).

**Figure 6.**
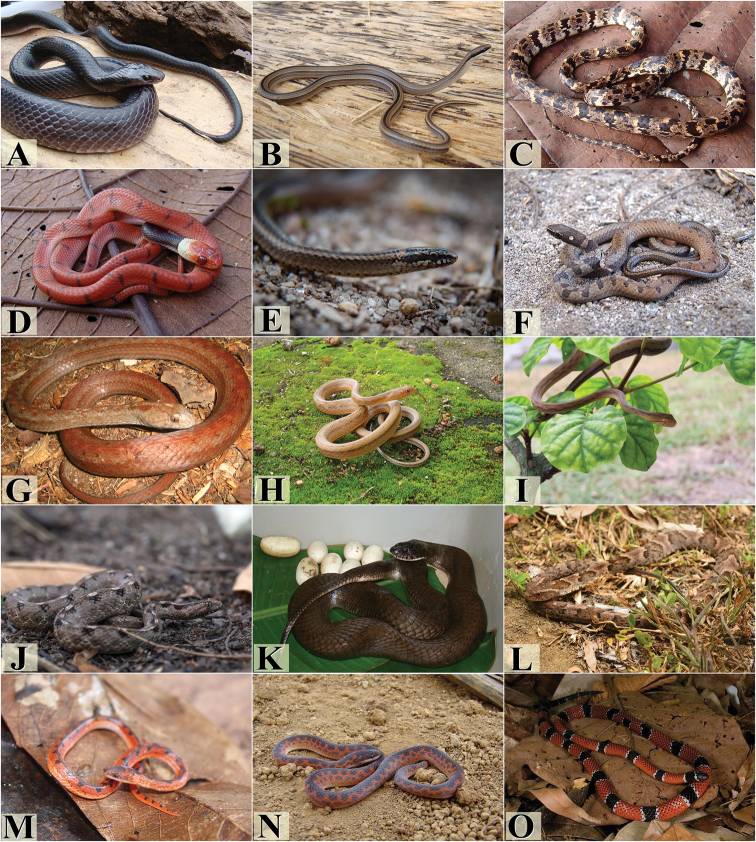
Snake species from the Pernambuco Endemism Center. **A***Pseudoboa nigra***B***Psomophis joberti*, **C***Sibon nebulatus***D***Siphlophis compressus***E***Taeniophallus affinis***F***Taeniophallus occipitalis***G***Thamnodynastes almae***H***Thamnodynastes hypoconia***I***Thamnodynastes pallidus***J***Thamnodynastes phoenix***K***Xenodon merremii***L***Xenodon rabdocephalus***M***Xenopholis scalaris***N***Xenopholis undulatus***O***Micrurus corallinus*. Photograph credits: Frederico França (**A, B, C, D, F, H, K, N**), Vanessa Nascimento (**L**), Samuel Cardoso (**G**), Davi Pantoja (**M**), Rafaela França **(I**), Anderson A. Santos (**E**), Paulo R. S. Freitas (**J**), Adrian Garda (**O**).

**Figure 7.**
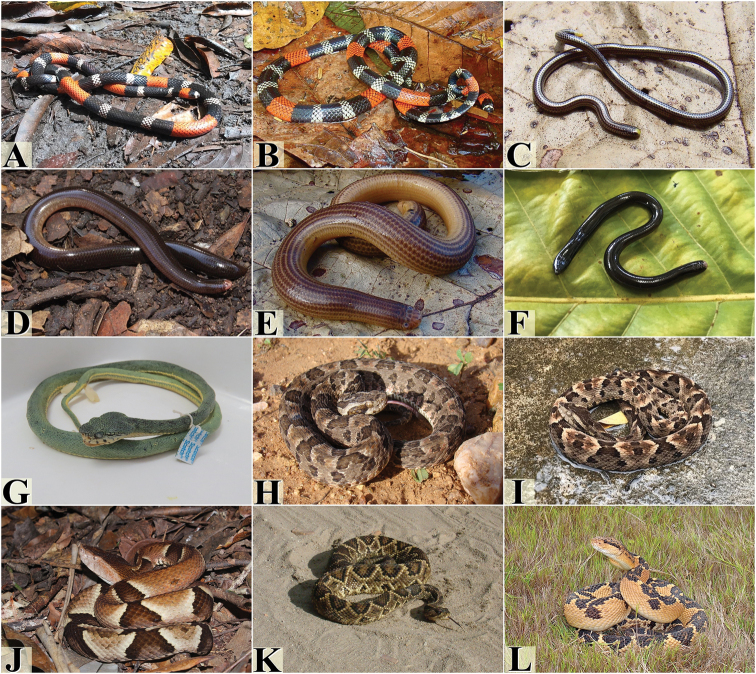
Snake species from the Pernambuco Endemism Center. **A***Micrurus ibiboboca***B***Micrurus potyguara***C***Epictia borapeliotes***D***Amerotyphlops arenensis***E***Amerotyphlops brongersmianus***F***Amerotyphlops paucisquamus***G***Bothrops bilineatus***H***Bothrops erythromelas***I***Bothrops leucurus***J***Bothrops muriciensis***K***Crotalus durissus***L***Lachesis muta*. Photograph credits: Frederico França (**A, B, E, F, H, I, K, L**), Ivan L. Sampaio (**C**), Gentil A. Pereira Filho (**D**), Willianilson Pessoa (**J**), Rafaela França (**G**).

We registered a total of 78 species of snakes of eight families, distributed in the PEC (Table 1, Figs 3–7). The most species rich family was Dipsadidae (47 species, 60% of total), followed by Colubridae (12 species, 15.4%), Viperidae (6 species, 7.7%), Boidae and Typhlopidae, both with four species (5.1%), Elapidae (3 species, 3.8 %) and Anomalepididae and Leptotyphlopidae, both with a single species (1.3%).

Many species of snakes that are found in PEC are also found in other Brazilian biomes. The Caatinga (58 species, 74.3% found in PEC) is the Brazilian biome that shares most species with the PEC, followed by Cerrado (44 species, 56.4%), Amazon Forest (35 species, 44.9%), Pantanal (35 species, 44.9%) and Pampas (13 species, 16.6%). On the other hand, some species (*Atractus caete*, *A. maculatus*, *Bothrops muriciensis*, *Caaeteboia* sp., *Dendrophidion atlantica*, *Echinanthera cephalomaculata* and *Micrurus potyguara*) are found only in the PEC and are considered endemic of this region.

Most of the snake species in the PEC have been registered in Forest areas (74 species, 94.8%), followed by Brejos Nordestinos (36 species, 46.1%), Tabuleiros (34 species, 43.5%), Restingas (11 species, 14.1%) and Mangroves (4 species, 5.1%). Six species were found in four different habitats and 31 species were found only in one habitat type (Table 1). Of these, 26 species were collected only in forested areas, three species only in the Brejos Nordestinos and one species was found only in restingas (Table 1).

**Table 1.** Summary of the Information of Natural History of the Snakes in the Pernambuco Endemism Center. Abbreviations are: Habitats (BN = Brejos Nordestinos, F = forest, Tb = Tabuleiro, Rt = Restinga, Mg = Mangrove); Diet (abn = amphisbaenians, amp = amphibians, ann = annelids, art = arthropods, bi = birds, fi = fishes, mo = mollusks, li = lizards, mam = mammals, sn=snakes; Activity period (D = Diurnal, N = Nocturnal); Habits (AB = arboreal, SAB = semi-arboreal, AQ = aquatic, SAQ = semi-aquatic, CR = cryptozoic, FS = Fossorial, TE = terrestrial). * Endemic species of the Pernambuco Endemism Center (PEC).

Family/Species	Habitats	Diet	Habits	Diel activity
** Anomalepididae **				
*Liotyphlops trefauti*	F	art	FS	N
** Boidae **				
*Boa constrictor*	BN, F, Tb, Rt	mam, li, bi	SAB, TE	D, N
*Corallus hortulanus*	F	mam, bi, li, amp	AB	N
*Epicrates assisi*	BN, F, Tb	mam, li, bi	TE	N
*Epicrates cenchria*	F	mam, bi, li, amp	TE, SAB	N
** Colubridae **				
*Chironius carinatus*	F	amp, bi, li, mam	TE, AB	D
*Chironius exoletus*	BN, F, Tb	amp, li	AR, TE	D
*Chironius flavolineatus*	BN, F, Tb	amp	SAB	D
Dendrophidion atlantica*	F	–	TE	D
*Drymarchon corais*	F, Tb	amp, abnli, sn, bi, mam	TE	D
*Drymoluber dichrous*	BN, F, Tb	li, amp	TE	D
*Leptophis ahaetulla*	BN, F	amp, li	AB,TE	D
*Oxybelis aeneus*	BN, F, Tb	li, amp, fi	AB	D
*Palusophis bifossatus*	F, BN	amp, mam, li	TE	D
*Spilotes pullatus*	BN, F, Tb	mam, bi	SAB	D
*Spilotes sulphureus*	F	mam, bi	SAB	D
*Tantilla melanocephala*	BN, F, Tb, Rt	art	FS	D, N
** Dipsadidae **				
*Apostolepis cearensis*	F, Tb	sn, abn	FS	D
*Apostolepis longicaudata*	F	sn	FS	D
Atractus caete*	F	ann	FS	N
*Atractus maculatus*	F	ann	FS	N
*Atractus potschi*	F	ann	FS	N
*Boiruna sertaneja*	Tb, F	sn, li, mam	TE	N
*Caaeteboia* sp.*	F	–	TE	D
*Dipsas mikanii*	BN, F, Tb	mo	TE	N
*Dipsas neuwiedi*	F, BN	mo	TE	N
*Dipsas sazimai*	F	mo	AB, TE	N
*Dipsas variegata*	F	mo	AB, TE	N
Echinanthera cephalomaculata*	F	amp	TE	D
*Echinanthera cephalostriata*	F	amp	TE	D
*Erythrolamprus aesculapii*	F	sn, li	TE	D
*Erythrolamprus almadensis*	F	amp	SAQ	D
*Erythrolamprus miliaris*	F, BN	amp, fi	SAQ	D, N
*Erythrolamprus poecilogyrus*	BN, F, Tb, Mg	amp, li	TE	D, N
*Erythrolamprus reginae*	F	amp, li, fi	SAQ	D
*Erythrolamprus taeniogaster*	F, Tb, Rt	amp, fi	SAQ	D
*Erythrolamprus viridis*	BN, F	amp, li	TE	D
*Helicops angulatus*	F, Mg, Rt	fi, amp	AQ	N
*Helicops leopardinus*	Rt, F	fi, amp	AQ	N
*Hydrodynastes gigas*	F, Rt	amp, fi, sn, mam	AQ, TE	D
*Imantodes cenchoa*	F, Tb	li, amp	AB	N
*Leptodeira annulata*	F, Rt, BN	amp, li	AB, TE	N
*Lygophis dilepis*	BN, F	amp	TE	D
*Oxyrhopus guibei*	BN, F, Tb	mam, li	TE	D, N
*Oxyrhopus petolarius*	BN, F, Tb	li, mam, bi, amp	TE	N
*Oxyrhopus trigeminus*	BN, F, Tb, Rt,	li, mam, bi	TE	D, N
*Philodryas nattereri*	BN, F, Tb	li, mam, amp, sn, bi	TE, SAB	D
*Philodryas olfersii*	BN, F, Tb, Mg	amp, li, bi, mam	TE, SAB	D
*Philodryas patagoniensis*	F, Tb, Rt	amp, li, mam, bi, sn	TE	D
*Phimophis guerini*	F, Tb	li, mam	TE	N
*Pseudoboa nigra*	BN, F, Tb	li, mam, sn	TE	N
*Psomophis joberti*	F	amp, li	TE	D
*Sibon nebulatus*	F, Tb	mo	AB	N
*Siphlophis compressus*	F, Tb	li, sn	AB, TE	N
*Taeniophallus affinis*	BN, F, Tb	li, amp, abn, mam	CR	N
*Taeniophallus occipitalis*	BN, F, Tb	li, amp, abn	CR	N
*Thamnodynastes almae*	BN	amp, li	AB, TE	N
*Thamnodynastes hypoconia*	BN	amp, li	TE, AB	N
*Thamnodynastes pallidus*	F, Tb	amp	TE, AB	N
*Thamnodynastes phoenix*	BN	amp	TE, AB	N
*Xenodon merremii*	BN, F, Tb	amp	TE	D
*Xenodon rabdocephalus*	F	amp	TE	D
*Xenopholis scalaris*	F	amp	TE	N
*Xenopholis undulatus*	BN, F	amp	TE	N
** Elapidae **				
*Micrurus corallinus*	F	abn, li, sn, amp	CR	D
*Micrurus ibiboboca*	BN, F, Tb	abn, sn, li	CR	D, N
Micrurus potyguara*	F, Tb	sn	CR	D, N
** Leptotyphlopidae **				
*Epictia borapeliotes*	F, BN, Rt	art	FS	D, N
** Typhlopidae **				
*Amerotyphlops amoipira*	Rt	art	FS	N
*Amerotyphlops arenensis*	BN, F	art	FS	N
*Amerotyphlops brongersmianus*	F, Tb	art	FS	N
*Amerotyphlops paucisquamus*	F, Tb	art	FS	N
** Viperidae **				
*Bothrops bilineatus*	F	mam, amp, bi, sn, li	AB	N
*Bothrops erythromelas*	F	li, mam	TE	N
*Bothrops leucurus*	F, BN, Tb, Mg	amp, li, sn, bi, mam	TE	N
Bothrops muriciensis*	F	amp, mam	TE	N
*Crotalus durissus*	BN, F, Rt	mam	TE	N
*Lachesis muta*	F	mam	TE	N

The majority of snake species found in the PEC use the soil as substrate, of which 47 species (60.2%) are terrestrial and 17 (21.7%) are cryptozoic and/or fossorial. In addition, 23 species are arboreal or semi-arboreal (29.5%) and 16 (20.5%) are aquatic or semi-aquatic. The diet of PEC snakes consists mainly of vertebrates (61 species, 78.2%), of which 23 species are considered generalists, feeding on three or more types of prey, 21 species feed on two types of prey, 23 species are specialists in amphibians, two species are specialists in snakes and two species are specialists in mammals. Only 14 species feed on invertebrates, of which six species feed on arthropods, three species feed on annelids and five species feed on mollusks (Table 1). As for the period of activity, 39 (50 %) species are nocturnal, 30 (38.4%) species are diurnal and nine (11.5%) species are diurnal and nocturnal (Table 1).

We present below a commented list of species of snakes that occur in PEC, with notes on natural history and distribution. The “N” corresponds to the number of individuals analyzed in the scientific collections. The species *L. trefauti*, *A. caete*, *A. potschi*, *E. cephalomaculata*, *E. cephalostriata*, *T. almae*, *T. hypoconia*, and *T. phoenix* were recorded only by literature data.

## Commented list

### Family Anomalepididae Taylor, 1939

**Figure 8.**
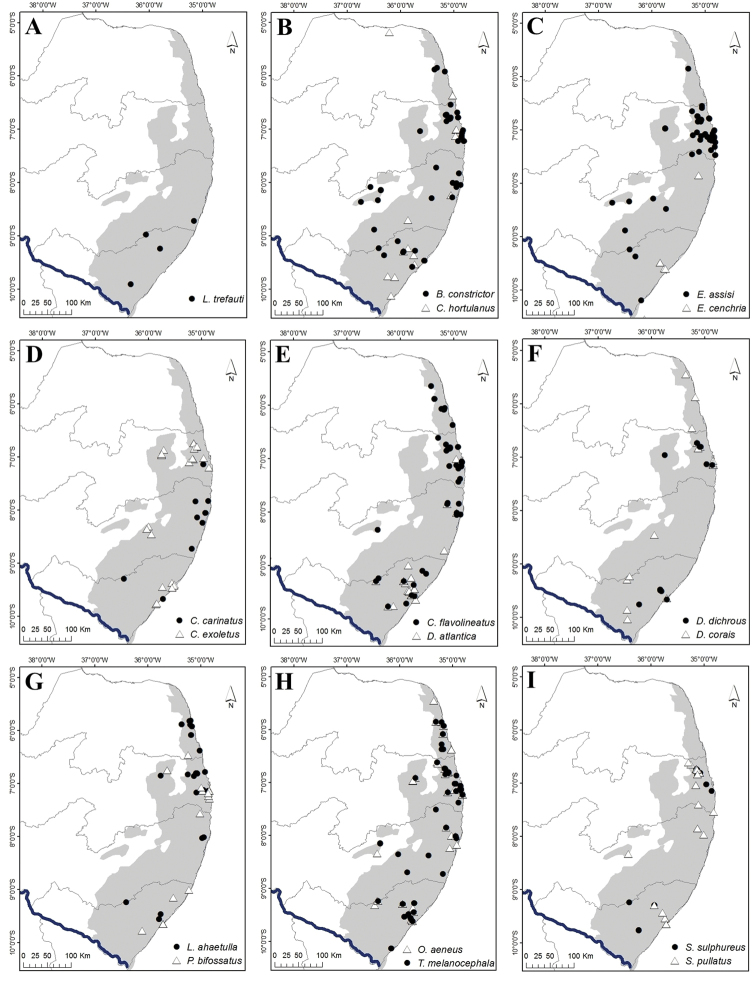
Geographic distribution records for snakes of the Pernambuco Endemism Center (PEC). **A***Liotyphlops trefauti***B***Boa constrictor* and *Corallus hortulanus***C***Epicrates cenchria* and *E. assisi***D***Chironius carinatus* and *C. exoletus***E***Chironius flavolineatus* and *Dendrophidion atlantica***F***Drymarchon corais* and *Drymoluber dichrous***G***Leptophis ahaetulla* and *Palusophis bifossatus***H***Oxybelis aeneus* and *Tantilla melanocephala***I***Spilotes sulphureus* and *S. pullatus*.

*Liotyphlops trefauti* Freire, Caramaschi, Suzart & Argolo, 2007 - A small-sized fossorial species (total length = 366–389 mm; *N* = 3), with nocturnal activity (Freire et al. 2007). It has a restricted distribution, occurring in the Atlantic Forest and Caatinga (Abegg et al. 2017b). In the PEC it occurs in the states of Alagoas and Pernambuco (Fig. 8A), being found in Forest areas (Freire et al. 2007, Abegg et al. 2017b). *Liotyphlops trefauti*, as observed in other congeneric species, feeds on eggs and arthropod larvae (Marques et al. 2019).

### Family Boidae Gray, 1825

*Boa constrictor* Linnaeus, 1758 - A large semiarboreal species (average SVL = 1023 mm; *N* = 42), with nocturnal activity (Marques et al. 2001). It has a wide distribution, occurring in the Atlantic Forest, Amazon Forest, Caatinga, Cerrado and Pantanal (Cunha and Nascimento 1993, Marques et al. 2005, 2015, 2019, Guedes et al. 2014). In the PEC it occurs in all states (Fig. 8B), being found in Forest, Brejos Nordestinos, Tabuleiros and Restinga Areas (Pereira Filho and Montingelli 2011, Rodrigues et al. 2015, Pereira Filho et al. 2017, Sampaio et al. 2018). This species can also occur in urban areas (França and França 2019). *Boa constrictor* feeds on mammals, birds and lizards (Pizzatto et al. 2010). Its litter can range from 18 to 60 hatchlings (Vitt and Vangilder 1983, Pizzatto and Marques 2007, Fraga et al. 2013).

*Corallus hortulanus* (Linnaeus, 1758) - A moderate-sized arboreal snake (SVL = 745 mm; *N* = 11), with nocturnal activity (Marques et al. 2019). It has a wide distribution, occurring in the Atlantic Forest, Amazon Forest, Caatinga, Cerrado and Pantanal (Marques et al. 2005, 2015, 2019, Fraga et al. 2013, Guedes et al. 2014). In the PEC it occurs in all states (Fig. 8B), being found in Forest. *Corallus hortulanus* feeds on mammals, birds, lizards and amphibians (Pizzatto et al. 2010). Its litter can range from 3 to 24 hatchlings (Pizzatto and Marques 2007, Fraga et al. 2013).

*Epicrates assisi* Machado, 1945 – A moderate-sized terrestrial species (average SVL = 691 mm; *N* = 135), with nocturnal activity (Marques et al. 2019). This species occurs in the Cerrado, Caatinga and Atlantic Forest (Guedes et al. 2014, Marques et al. 2015, 2019). In the PEC it occurs in all states (Fig. 8C), being found in Forest, Brejos Nordestinos, Tabuleiros, Restingas and urban areas (França et al. 2012, Rodrigues et al. 2015, Pereira Filho et al. 2017, Sampaio et al. 2018). *Epicrates assisi* feeds on mammals, birds, and lizards. Its litter can range from 7 to 14 hatchlings (Pizzatto and Marques 2007).

*Epicrates cenchria* (Linnaeus, 1758) – A large semi-arboreal or terrestrial species (average SVL = 1105 mm; *N* = 6), with nocturnal activity (Marques et al. 2019). It has a wide distribution, occurring in the Atlantic Forest, Amazon Forest, Cerrado and Pantanal (Marques et al. 2005, 2015, 2019, Passos and Fernandes 2008). In the PEC it occurs in the states of Alagoas and Pernambuco (Fig. 8C), being found in Forest areas, but also in urban areas. *Epicrates cenchria* feeds on mammals, birds, lizards and amphibians (Martins and Oliveira 1998, Pizzatto et al. 2010). Its litter can range from 8 to 25 hatchlings (Pizzatto and Marques 2007).

### Family Colubridae Oppel, 1811

*Chironius carinatus* (Linnaeus, 1758) – A large terrestrial and arboreal species (average SVL = 1001 mm; *N* = 15), with diurnal activity (Marques et al. 2019). It has a disjunct distribution, occurring in the Amazon Forest and Atlantic Forest (Araújo et al. 2019). In the PEC it occurs in the states of Alagoas, Pernambuco and Paraíba (Fig. 8D), being found in Forest and urban areas when these are close to forests (Araújo et al. 2019). *Chironius carinatus* feeds on amphibians, birds, lizards and mammals (Dixon et al. 1993, Silva et al. 2010, Rodrigues et al. 2016). Its litter can have 5 to 12 eggs (Dixon et al. 1993, Goldberg 2007).

*Chironius exoletus* (Linnaeus, 1758) – A moderate-sized arboreal and terrestrial species (average SVL = 614 mm; *N* = 16), with diurnal activity (Marques et al. 2019). It has a wide distribution, occurring in the Atlantic Forest, Caatinga, Cerrado, Pantanal and Amazon Forest (Cunha and Nascimento 1993, Marques et al. 2005, 2015, 2019, Guedes et al. 2014). In the PEC it occurs in the states of Alagoas, Pernambuco and Paraíba (Fig. 8D), being found in Forest, Brejos Nordestinos and Tabuleiro (Pereira Filho and Montingelli 2011, Rodrigues et al. 2015). *Chironius exoletus* feeds mainly on amphibians, but occasionally on lizards (Marques and Sazima 2004, Rodrigues et al. 2016). Its litter can range from 4 to 12 eggs (Dixon et al. 1993, Goldberg 2007).

*Chironius flavolineatus* (Linnaeus, 1758) – A moderate-sized semi-arboreal species (average SVL = 592 mm; *N* = 60), with diurnal activity (Marques et al. 2019). It presents a wide distribution, occurring in the Atlantic Forest, Cerrado, Caatinga, Pantanal and Amazon Forest (Cunha and Nascimento 1993, Dixon et al. 1993, Marques et al. 2005, 2015, Guedes et al. 2014). In the PEC it occurs in all states (Fig. 8E), being found in Forest, Brejos Nordestinos, Tabuleiros, and urban areas (França et al. 2012, Rodrigues et al. 2015, Sampaio et al. 2018). *Chironius flavolineatus* feeds on amphibians (Pinto et al. 2008, Rodrigues et al. 2016). Its litter can range from 3 to 8 eggs (Dixon et al. 1993, Hamdan and Fernandes 2015).

*Dendrophidion atlantica* Freire, Caramaschi & Gonçalves, 2010 – A small-sized terrestrial species (average SVL = 366 mm; *N* = 24), with diurnal activity (Marques et al. 2019). *Dendrophidion atlantica* is endemic to the PEC and occurs in the states of Alagoas, Pernambuco and Paraíba (Fig. 8E), being found in Forest (Freire et al. 2010, Pereira Filho et al. 2017, Barbosa et al. 2019). *Dendrophidion atlantica* feeds on amphibians (Marques et al. 2019). Its litter can have 3 eggs (Lima et al. 2019).

*Drymarchon corais* (Boie, 1827) – A large terrestrial species (average SVL = 1288 mm; *N* = 7), with diurnal activity (Marques et al. 2019). It presents a wide distribution, being registered in the Amazon Forest, Cerrado, Caatinga and Pantanal (Cunha and Nascimento 1993, Strussmann and Sazima 1993, Guedes et al. 2014, Marques et al. 2015, 2019). In the PEC it occurs in all states (Fig. 8F), being found in Forest, Tabuleiros and urban areas (Rodrigues et al. 2015, Mesquita et al. 2018). *Drymarchon corais* feeds on amphibians, amphisbaenians, lizards, snakes, birds and mammals (Prudente et al. 2014). Its litter can range from 3 to 15 eggs (Prudente et al. 2014).

*Drymoluber dichrous* (Peters, 1863) – A small-sized terrestrial species (average SVL = 348 mm; *N* = 15), with diurnal activity (Marques et al. 2019). This species occurs in the Atlantic Forest, Amazon Forest, and Caatinga (Cunha and Nascimento 1993, Guedes et al. 2014, Marques et al. 2019). In the PEC it occurs in the states of Alagoas and Paraíba (Fig. 8F), being found in Forest, Brejos Nordestinos, Tabuleiros and urban areas (Rodrigues et al. 2015, Pereira Filho et al. 2017, Mesquita et al. 2018, França and França 2019). *Drymoluber dichrous* feeds on lizards and amphibians (Martins and Oliveira 1998, Borges-Nojosa and Lima 2001). Its litter can range from 2 to 6 eggs (Martins and Oliveira 1998, Fraga et al. 2013).

*Leptophis ahaetulla* (Linnaeus, 1758) – An arboreal and terrestrial, moderate-sized species (average SVL = 582 mm; *N* = 42), with diurnal activity (Marques et al. 2019). This species occurs in Atlantic Forest, Amazon Forest, Caatinga, Cerrado, Pantanal, and Pampas (Strussmann and Sazima 1993, Bérnils et al. 2007, Guedes et al. 2014, Marques et al. 2015, 2019). In the PEC it can be found in all states (Fig. 8G) in Forest, Brejos Nordestinos and urban areas (Pereira Filho and Montingelli 2011, França and França 2019). *Leptophis ahaetulla* feeds on amphibians and lizards (Albuquerque et al. 2007). Its litter can range from 3 to 12 eggs (Vitt and Vangilder 1983, Mesquita et al. 2009).

*Oxybelis aeneus* (Wagler, 1824) – An arboreal, moderate-sized species (average SVL = 780 mm; *N* =46), with diurnal activity (Marques et al. 2019). It presents a wide distribution, being found in the Atlantic Forest, Amazon Forest, Caatinga, Cerrado, and Pantanal (Cunha and Nascimento 1993, Marques et al. 2005, 2015, 2019, Guedes et al. 2014). In the PEC it occurs in all states (Fig. 8H), being found in Forest, Brejos Nordestinos, Tabuleiros, and urban areas (Pereira Filho and Montingelli 2011, Rodrigues et al. 2015, França and França 2019). *Oxybelis aeneus* feeds on lizards, amphibians, and occasionally fishes (Henderson 1982, Hetherington 2006, Grant and Lewis 2010, Mesquita et al. 2013, Franzini et al. 2018). Its litter can range from 4 to 9 eggs (Vitt and Vangilder 1983, Mesquita et al. 2009, Fraga et al. 2013).

*Palusophis bifossatus* (Raddi, 1820) – A moderate-sized terrestrial species (average SVL = 801 mm; *N* = 5), with diurnal activity (Marques et al. 2019). It presents a wide distribution, occurring in the Atlantic Forest, Amazon Forest, Caatinga, Cerrado, Pampas, and Pantanal (Cunha and Nascimento 1993, Strussmann and Sazima 1993, Lema 2003, Bérnils et al. 2007, Guedes et al. 2014, Marques et al. 2019). In the PEC it occurs in all states (Fig. 8G), being found in Forest and Brejos Nordestinos (Pereira Filho and Montingelli 2011, Pereira Filho et al. 2017). *Palusophis bifossatus* feeds on amphibians, mammals, and lizards (Leite et al. 2007). Its litter can range from 4 to 24 eggs (Costa et al. 2010).

*Spilotes pullatus* (Linnaeus, 1758) – A large, semi-arboreal species (average SVL = 1442 mm; *N* = 21), with diurnal activity (Marques et al. 2019). It presents a wide distribution, being found in the Atlantic Forest, Amazon Forest, Caatinga, Cerrado, and Pantanal (Cunha and Nascimento 1993, Marques et al. 2005, 2015, 2019, Guedes et al. 2014). In the PEC, it occurs in the states of Alagoas, Pernambuco and Paraíba (Fig. 8I), being found in Forest, Brejos Nordestinos, Tabuleiros, and urban areas (Pereira Filho et al. 2017, Mesquita et al. 2018, França and França 2019). *Spilotes pullatus* feeds on mammals and birds (Silva et al. 2010, Marques et al. 2014). Its litter can range from 2 to 5 eggs (Hauzman et al. 2005, Fraga et al. 2013).

*Spilotes sulphureus* (Wagler, 1824) – A moderate-sized semi-arboreal species (average SVL = 911 mm; *N* =20), with diurnal activity (Marques et al. 2019). It presents a wide distribution, being found in the Atlantic Forest, Amazon Forest, Caatinga and Cerrado (Cunha and Nascimento 1993, Guedes et al. 2014, Marques et al. 2015, 2019). In the PEC, it occurs in the states of Alagoas and Paraíba (Fig. 8I), being found in Forest and urban areas (Morais et al. 2018). *Spilotes sulphureus* feeds on mammals and birds (Beebe 1946, Cunha and Nascimento 1993, Rufino and Bernardi 1999). Its litter can range from 7 to 15 eggs (Good 1989, Fraga et al. 2013, Morais et al. 2018).

*Tantilla melanocephala* (Linnaeus, 1758) – A small-sized fossorial species (average SVL = 233 mm; *N* = 172), with diurnal and nocturnal activity (Marques et al. 2019). It presents a wide distribution, occurring in the Atlantic Forest, Amazon Forest, Caatinga, Cerrado, Pampas, and Pantanal (Cunha and Nascimento 1993, Marques et al. 2005, 2015, 2019, Bérnils et al. 2007, Guedes et al. 2014). In the PEC it occurs in all states (Fig. 8H), being found in Forest, Brejos Nordestinos, Tabuleiros, and restingas (Pereira Filho and Montingelli 2011, Mesquita et al. 2018, Sampaio et al. 2018). *Tantilla melanocephala* feeds on arthropods. Its litter can range from 1 to 3 eggs (Mesquita et al. 2009, Fraga et al. 2013)

### Dipsadidae Bonaparte, 1838

**Figure 9.**
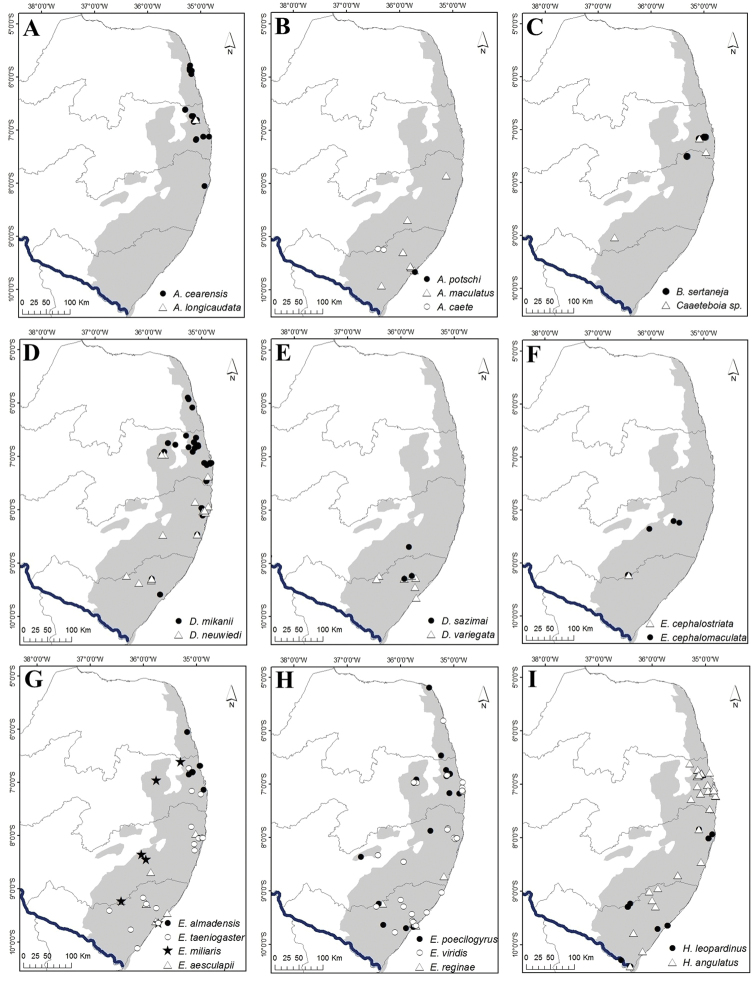
Geographic distribution records for snakes of the Pernambuco Endemism Center (PEC). **A***Apostolepis longicaudata* and *A. cearensis***B***Atractus caete*, *A. maculatus* and *A. potschi***C***Boiruna sertaneja* and *Caaeteboia* sp. **D***Dipsas mikanii* and *D. neuwiedi***E***D. sazimai* and *D. variegata***F***Echinanthera cephalomaculata* and *E. cephalostriata***G***Erythrolamprus almadensis*, *E. taeniogaster*, *E. miliaris*, and *E. aesculapii***H***E. poecilogyrus*, *E. viridis* and *E. reginae*. **I***Helicops angulatus* and *H. leopardinus*.

*Apostolepis cearensis* Gomes, 1915 – A small-sized fossorial species (average SVL = 329 mm; *N* = 44), with diurnal activity (Marques et al. 2019). This species occurs in the Atlantic Forest, Caatinga and Cerrado (Guedes et al. 2014, Marques et al. 2015, Mesquita et al. 2018). In the PEC it occurs in the states of Rio Grande do Norte, Paraíba and Pernambuco (Fig. 9A), being found in Forest, Tabuleiros, and urban areas (Mesquita et al. 2018, França and França 2019). *Apostolepis cearensis* feeds on small elongated reptiles (Mesquita et al. 2009, Amorim et al. 2015, Marques et al. 2019).

*Apostolepis longicaudata* Gomes, 1921 – A small-sized fossorial species (average SVL = 235 mm; *N* = 8), with diurnal activity (Marques et al. 2019). This species occurs in the Cerrado, Caatinga and Floresta Atlântica (Curcio et al. 2011, França et al. 2012). In the PEC it occurs only in a conservation unit (Reserva Biológica Guaribas) located in the state of Paraíba (Fig. 9A), being found in Forest. *Apostolepis longicaudata* feeds on small elongated reptiles (Marques et al. 2019). We found two eggs in a female.

*Atractus caete* Passos, Fernandes, Bérnils & Moura-Leite, 2010 – A small-sized fossorial and cryptozoic species (average SVL = 376 mm, *N* = 1), with nocturnal activity (Passos et al. 2010, Marques et al. 2019). This species is endemic to the PEC and occurs only in the state of Alagoas (Fig. 9B), being found in Forest areas. *Atractus caete* feeds mostly on earthworms (Passos et al. 2010).

*Atractus maculatus* (Günther, 1858) – A small-sized fossorial and cryptozoic species (average SVL = 326 mm; *N* = 5), with nocturnal activity (Marques et al. 2019). This species occurs in the Atlantic Forest and Caatinga (Passos et al. 2010, Abegg et al. 2017a). In the PEC it occurs in the states of Alagoas and Pernambuco (Fig. 9B), being found in Forest and urban areas, when close to forests. *Atractus maculatus* feeds mostly on earthworms (Passos et al. 2010).

*Atractus potschi* Fernandes, 1995 – A small-sized fossorial and cryptozoic species (average SVL = 312 mm, *N* = 1), with nocturnal activity (Passos et al. 2010, Marques et al. 2019). This species occurs in the Atlantic Forest and Caatinga (Guedes et al. 2014). In the PEC it occurs in the state of Alagoas (Fig. 9B), being found in Forest (Passos et al. 2010). *Atractus potschi* feeds mostly on earthworms (Passos et al. 2010).

*Boiruna sertaneja* Zaher, 1996 – A large terrestrial species (average SVL = 1358 mm; *N* = 2), with nocturnal activity (Marques et al. 2019). This species occurs in the Atlantic Forest and Caatinga (Guedes et al. 2014, Pereira Filho et al. 2017). In the PEC it can be found in the states of Pernambuco and Alagoas (Fig. 9C), in Tabuleiros and Forest (Rodrigues et al. 2015, Pereira Filho et al. 2017). *Boiruna sertaneja* eats snakes, lizards and mammals (Vitt and Vangilder 1983, Gaiarsa et al. 2013). Its litter can range from 4 to 14 eggs (Vitt and Vangilder 1983, Gaiarsa et al. 2013).

*Caaeteboia* sp. – A small to moderate-sized terrestrial species (average SVL = 411 mm; *N* = 2), with diurnal activity (personal observation). This species is endemic to the PEC and occurs only in the states of Pernambuco and Paraíba (Fig. 9C), being found in Forest.

*Dipsas mikanii* Schlegel, 1837 – A small-sized terrestrial species (average SVL = 302 mm; *N* = 72), with nocturnal activity (Marques et al. 2019). This species occurs in the Atlantic Forest, Cerrado, Caatinga and Pantanal (Marques et al. 2005, 2015, 2019, Guedes et al. 2014). In the PEC it occurs in all states (Fig. 9D), being found in Forest, Brejos Nordestinos, Tabuleiros and urban areas (França et al. 2012, Pereira Filho et al. 2017, Sampaio et al. 2018). *Dipsas mikanii* feeds on mollusks (Laporta-Ferreira et al. 1986). Its litter can range from 3 to 10 eggs (Pizzatto et al. 2008).

*Dipsas neuwiedi* (Ihering, 1911) – A small-sized terrestrial species (average SVL = 369 mm; *N* = 17), with nocturnal activity (Marques et al. 2019). This species occurs in the Atlantic Forest and Caatinga (Guedes et al. 2014, Marques et al. 2019). In the PEC it occurs in the states of Alagoas, Pernambuco and Paraíba (Fig. 9D), being found in Forest, Brejos Nordestinos and urban areas (Pereira Filho et al. 2017). *Dipsas neuwiedi* feeds on mollusks (Laporta-Ferreira et al. 1986). Its litter can range from 4 to 12 eggs (Pizzatto et al. 2008).

*Dipsas sazimai* Fernandes, Marques & Argôlo, 2010 – A small-sized arboreal and terrestrial species (average SVL = 299 mm; *N* = 1), with nocturnal activity (Marques et al. 2019). This species occurs in the Atlantic Forest and Caatinga (Fernandes et al. 2010, Guedes et al. 2014). In the PEC it occurs in the states of Alagoas and Pernambuco (Fig. 9E), being found in Forest. *Dipsas sazimai* feeds on mollusks (Fernandes et al. 2010).

*Dipsas variegata* (Duméril, Bibron & Duméril, 1854) – A small to moderate size arboreal and terrestrial species (average SVL = 464 mm; *N* = 4), with nocturnal activity (Marques et al. 2019). This species occurs in the Atlantic Forest and Amazon Forest (Cunha and Nascimento 1993, Marques et al. 2019). In the PEC it occurs only in the state of Alagoas (Fig. 9E), being found in Forest. *Dipsas variegata* feeds on mollusks (Marques et al. 2019).

*Echinanthera cephalomaculata* Di Bernardo, 1994 – A small to moderate size terrestrial species (average SVL = 297 mm, *N* = 2), with diurnal activity (Di-Bernardo 1994, Marques et al. 2019). This species is endemic to the PEC and occurs only in the states of Alagoas and Pernambuco (Fig. 9F), being found in Forest (Roberto et al. 2015, Freitas et al. 2019b). *Echinanthera cephalomaculata* feeds on amphibians (Marques et al. 2019).

*Echinanthera cephalostriata* Di Bernardo, 1996 – A moderate-sized terrestrial species, with diurnal activity (Di-Bernardo 1996, Marques et al. 2019). This species only occurs in the Atlantic Forest (Marques et al. 2019). In the PEC it occurs in the state of Alagoas (Fig. 9F), being found only in the Reserva Biológica de Pedra Talhada (Roberto et al. 2015). In the report of this species for the PECRoberto et al. (2015) provide a photo and a voucher (URCA-H 4103). *Echinanthera cephalostriata* feeds on amphibians (Marques et al. 2009).

*Erythrolamprus aesculapii* (Linnaeus, 1758) – A moderate-sized terrestrial species (average SVL = 562 mm; *N* = 7), with diurnal activity (Marques et al. 2019). This species occurs in the Atlantic Forest, Amazon forest, Caatinga, Cerrado and Pantanal (Cunha and Nascimento 1993, Marques et al. 2005, 2015, 2017a, 2019). In the PEC it occurs in the states of Alagoas and Pernambuco (Fig. 9G), being found in Forest and urban areas. *Erythrolamprus aesculapii* feeds on snakes and lizards (Marques and Puorto 1992). Its litter can range from 1 to 8 eggs (Marques 1996a).

*Erythrolamprus almadensis* (Wagler, 1824) – A small-sized semi-aquatic species (average SVL = 298 mm; *N* = 4), with diurnal activity (Marques et al. 2019). This species has a wide distribution, occurring in the Atlantic Forest, Amazon forest, Caatinga, Cerrado, Pantanal and Pampas (Dixon 1989, França et al. 2006, Bérnils et al. 2007, Guedes et al. 2014). In the PEC it occurs in the states of Paraíba and Rio Grande do Norte (Fig. 9G), being found in Forest (Pereira Filho et al. 2017, França and França 2019). *Erythrolamprus almadensis* feeds on amphibians (Bernarde and Abe 2010, Rodrigues et al. 2016). Its litter can have five eggs.

*Erythrolamprus miliaris* (Linnaeus, 1758) – A small-sized semi-aquatic species (average SVL = 382 mm; *N* = 7), with diurnal and nocturnal activity (Marques et al. 2019). This species occurs in the Atlantic Forest, Amazon forest, Caatinga and Cerrado (Cunha and Nascimento 1993, Nogueira et al. 2010, Marques et al. 2017a, 2019). In the PEC it occurs in the states of Alagoas, Pernambuco and Paraíba (Fig. 9G), being found in Forest and Brejos Nordestinos. *Erythrolamprus miliaris* feeds on amphibians and fish (Marques et al. 2019). Its litter can range from 1 to 30 eggs (Pizzatto and Marques 2006).

*Erythrolamprus poecilogyrus* (Wied-Neuwied, 1825) – A small-sized terrestrial species (average SVL = 313 mm; *N* = 35), with diurnal and nocturnal activity (Marques et al. 2019). This species occurs in the Atlantic Forest, Caatinga, Cerrado, Pantanal and Pampas (Marques et al. 2005, 2015, 2019, Bérnils et al. 2007, Guedes et al. 2014). In the PEC it occurs in all states (Fig. 9H), being found in Forest, Brejos Nordestinos, Mangroves, Tabuleiros and urban areas (França et al. 2012, Pereira Filho et al. 2017, Mesquita et al. 2018). *Erythrolamprus poecilogyrus* feeds on amphibians and lizards (Prieto et al. 2012). Its litter can range from 3 to 17 eggs (Vitt and Vangilder 1983, Mesquita et al. 2009). In Figure 4N we show a juvenile that is in the process of changing its coloration to the adult stage. This species has a different color pattern in the region (Pereira Filho et al. 2017) if compared to other populations located more southwards.

*Erythrolamprus reginae* (Linnaeus, 1758) – A small-sized semi-aquatic species (average SVL = 355 mm; *N* = 4), with diurnal activity (Marques et al. 2019). This species occurs in the Atlantic and Amazon forests, Caatinga, Cerrado and Pantanal (Cunha and Nascimento 1993, Marques et al. 2005, 2015, 2019, Guedes et al. 2014). In the PEC it occurs in the states of Alagoas and Pernambuco (Fig. 9H), being found in Forest. *Erythrolamprus reginae* feeds on amphibians, lizards, and fish (Martins and Oliveira 1998, Albarelli and Santos-Costa 2010, Silva et al. 2010, Rodrigues et al. 2016). Its litter can range from 1 to 4 eggs (Arzamendia 2016, Marques et al. 2016)

*Erythrolamprus taeniogaster* (Jan, 1863) – A small-sized semi-aquatic species (average SVL = 364 mm; *N* = 45), with diurnal activity (Marques et al. 2019). This species occurs in the Atlantic Forest, Amazon forest, Caatinga, Cerrado and Pantanal (Cunha and Nascimento 1993, Marques et al. 2005, 2015, 2019, Guedes et al. 2014). In the PEC it occurs in the states of Alagoas, Pernambuco and Paraíba (Fig. 9G), being found in Forest, Tabuleiros, Restingas and urban areas (Rodrigues et al. 2015, Pereira Filho et al. 2017, Mesquita et al. 2018, Sampaio et al. 2018). *Erythrolamprus taeniogaster* feeds on amphibians and fish (Cunha and Nascimento 1993, Rodrigues et al. 2016). Its litter can range from 7 to 10 eggs (Cunha and Nascimento 1993).

*Erythrolamprus viridis* (Günther, 1862)– A small-sized terrestrial species (average SVL = 243 mm; *N* = 21), with diurnal activity (Marques et al. 2019). This species occurs in the Atlantic Forest and Caatinga (Guedes et al. 2014, Marques et al. 2019). In the PEC it occurs in all states (Fig. 9H), being found in Forest, Brejos Nordestinos and urban areas (Pereira Filho and Montingelli 2011, Pereira Filho et al. 2017). *Erythrolamprus viridis* feeds on amphibians and lizards (Vitt and Vangilder 1983, Mesquita et al. 2009). Its litter can range from 2 to 7 eggs (Vitt and Vangilder 1983, Mesquita et al. 2009).

*Helicops angulatus* (Linnaeus, 1758) – A small to moderate sized aquatic species (average SVL = 413 mm; *N* = 236), with nocturnal activity (Marques et al. 2019). This species occurs in the Atlantic and Amazon forests, Caatinga, Cerrado and Pantanal (Cunha and Nascimento 1993, Marques et al. 2005, 2015, 2019, Guedes et al. 2014). In the PEC it occurs in all states (Fig. 9I), being found in Forest, Mangroves, Restingas and urban areas (França et al. 2012, Pereira Filho et al. 2017, Sampaio et al. 2018). *Helicops angulatus* feeds on fish and amphibians. Its litter can range from 1 to 21 eggs (Braz et al. 2016).

*Helicops leopardinus* (Schlegel, 1837) – A small-sized aquatic species (average SVL = 324 mm; *N* = 9), with nocturnal activity (Marques et al. 2019). This species occurs in the Atlantic Forest, Amazon Forest, Caatinga, Cerrado, Pantanal and Pampas (Strussmann and Sazima 1993, Marques et al. 2005, 2015, 2019, Bérnils et al. 2007, Guedes et al. 2014, Rodrigues et al. 2016). In the PEC it occurs in the states of Alagoas and Pernambuco (Fig. 9I), being found in Forest, Restingas and urban areas. *Helicops leopardinus* feeds on fish and amphibians (Ávila et al. 2006). Its litter can range from 3 to 31 eggs (Scartozzoni and Almeida-Santos 2006, Braz et al. 2016).

**Figure 10.**
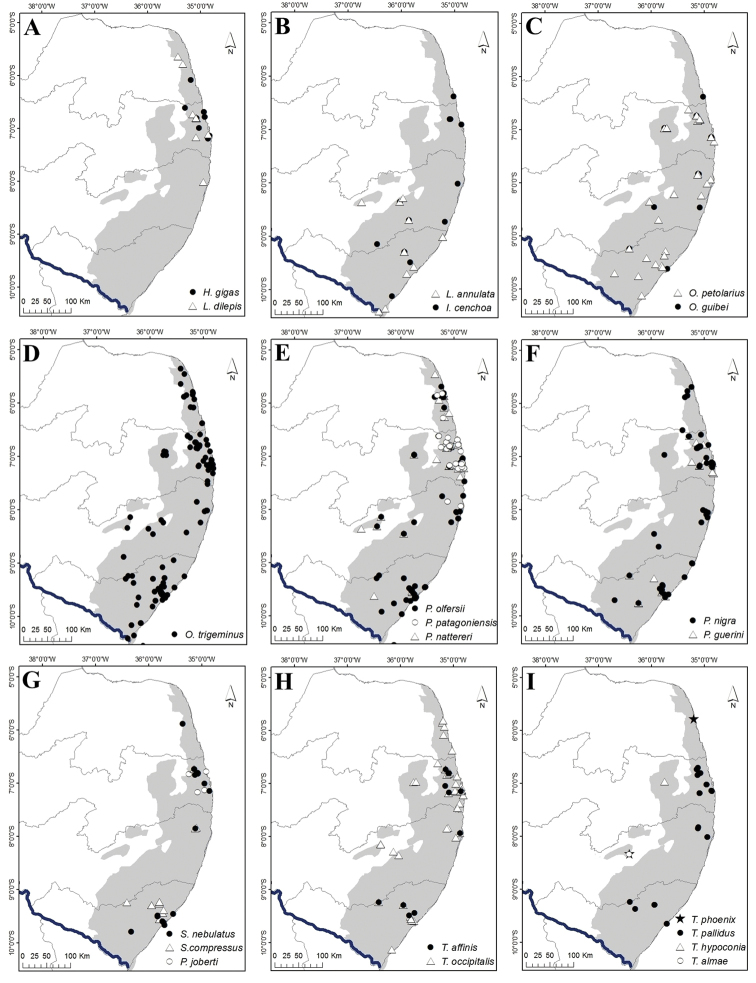
Geographic distribution records for snakes of the Pernambuco Endemism Center (PEC). **A***Hydrodynastes gigas* and *Lygophis dilepis***B***Imantodes cenchoa* and *Leptodeira annulata***C***Oxyrhopus guibei* and *O. petolarius***D***O. trigeminus***E***Philodryas nattereri*, *P. olfersii* and *P. patagoniensis***F***Phimophis guerini* and *Pseudoboa nigra***G***Psomophis joberti*, *Sibon nebulatus* and *Siphlophis compressus***H***Taeniophallus affinis* and *T. occipitalis***I***Thamnodynastes almae*, *T. hypoconia*, *T. pallidus* and *T. phoenix*.

*Hydrodynastes gigas* (Duméril, Bibron & Duméril, 1854) – A large aquatic and terrestrial species (average SVL = 1296 mm; *N* =10), with diurnal activity (Marques et al. 2019). This species occurs in the Atlantic Forest, Amazon Forest, Cerrado, Pantanal and Pampas (Lema 2003, Marques et al. 2005, 2015, 2019, Rodrigues et al. 2016). In the PEC it occurs in the states of Paraíba and Rio Grande do Norte (Fig. 10A), being found in Forest and Restingas (Pereira Filho et al. 2017, Sampaio et al. 2018). *Hydrodynastes gigas* feeds on fish, amphibians, mammals and snakes (López and Giraudo 2004). Its litter can range from 14 to 42 eggs (Vogel 1958, Fraga et al. 2013).

*Imantodes cenchoa* (Linnaeus, 1758) – An arboreal, moderate-sized species (average SVL = 633 mm; *N* = 23), with nocturnal activity (Marques et al. 2019). This species occurs in the Atlantic Forest, Amazon Forest, Caatinga, Cerrado and Pantanal (Cunha and Nascimento 1993, Marques et al. 2005, 2015, 2019, Guedes et al. 2014). In the PEC it occurs in all states (Fig. 10B), being found in Forest and Tabuleiros (Rodrigues et al. 2015, Mesquita et al. 2018). *Imantodes cenchoa* feeds on amphibians and lizards (Martins and Oliveira 1998, Sousa et al. 2014). Its litter can range from 1 to 7 eggs (Martins and Oliveira 1998, Pizzatto et al. 2008, Fraga et al. 2013, Sousa et al. 2014).

*Leptodeira annulata* (Linnaeus, 1758) – A moderate-sized arboreal and terrestrial species (average SVL = 576 mm; *N* = 6), with nocturnal activity (Marques et al. 2019). This species occurs in the Atlantic Forest, Amazon Forest, Caatinga, Cerrado and Pantanal (Ávila and Morais 2007, Guedes et al. 2014, Marques et al. 2015, 2019). In the PEC it occurs in the states of Alagoas and Pernambuco (Fig. 10B), being found in Forest, Brejos Nordestinos, and Restingas (Pereira Filho and Montingelli 2011, Roberto et al. 2015). *Leptodeira annulata* feeds on amphibians and lizards (Moura 1999, Mesquita et al. 2013, Santos-Silva et al. 2014). Its litter can range from 3 to 13 eggs (Petzold 1969, Pizzatto et al. 2008).

*Lygophis dilepis* Cope, 1862 – A small-sized terrestrial species (average SVL = 356 mm; *N* = 9), with diurnal activity (Marques et al. 2019). This species occurs in the Atlantic Forest, Caatinga and Cerrado (Guedes et al. 2014, Marques et al. 2015, Mesquita et al. 2018). In the PEC it occurs in the states of Pernambuco, Paraíba and Rio Grande do Norte (Fig. 10A), being found in Forest, Brejos Nordestinos, and urban areas (Pereira Filho and Montingelli 2011, França et al. 2012, Mesquita et al. 2018). *Lygophis dilepis* feeds on amphibians (Mesquita et al. 2009). Its litter can range from 4 to 6 eggs (Mesquita et al. 2009).

*Oxyrhopus guibei* Hoge & Romano, 1977 – A small sized terrestrial species (average SVL = 442 mm; *N* = 10), with diurnal and nocturnal activity (Marques et al. 2017). This species occurs in the Atlantic Forest, Caatinga, Cerrado and Pantanal (Marques et al. 2005, 2015, 2019, Guedes et al. 2014). In the PEC it occurs in all states (Fig. 10C), being found in Forest, Brejos Nordestinos, and Tabuleiros (Pereira Filho and Montingelli 2011, Mesquita et al. 2018). *Oxyrhopus guibei* feeds on mammals and lizards (Andrade and Silvano 1996, Barbo et al. 2011). Its litter can range from 3 to 20 eggs (Pizzatto and Marques 2002).

*Oxyrhopus petolarius* (Linnaeus, 1758) – A small size terrestrial species (average SVL = 423 mm; *N* = 36), with nocturnal activity (Marques et al. 2017). This species occurs in the Atlantic Forest, Amazon Forest, Caatinga, Cerrado and Pantanal (Marques et al. 2005, 2015, 2019, Guedes et al. 2014). In the PEC it occurs in all states (Fig. 10C), being found in Forest, Brejos Nordestinos, Tabuleiros, and urban areas (Pereira Filho et al. 2017, Mesquita et al. 2018, Sampaio et al. 2018, França and França 2019). *Oxyrhopus petolarius* feeds on lizards, mammals, birds and amphibians (Alencar et al. 2013). Its litter can range from 2 to 12 eggs (Lynch 2009, Gaiarsa et al. 2013).

*Oxyrhopus trigeminus* Duméril, Bibron & Duméril, 1854 – A small-sized terrestrial species (average SVL = 360 mm; *N* = 237), with nocturnal activity (Marques et al. 2017). This species occurs in the Atlantic Forest, Caatinga, Cerrado and Pantanal (Marques et al. 2005, 2015, 2019, Guedes et al. 2014). In the PEC it occurs in all states (Fig. 10D), being found in Forest, Brejos Nordestinos, Restingas, Tabuleiros, and urban areas (Pereira Filho and Montingelli 2011, Sampaio et al. 2018, França and França 2019). *Oxyrhopus trigeminus* feeds on lizards, mammals, and birds (Vitt and Vangilder 1983, Mesquita et al. 2009, Alencar et al. 2012). Its litter can range from 6 to 9 eggs (Vitt and Vangilder 1983, Mesquita et al. 2009).

*Philodryas nattereri* Steindachner, 1870 – A moderate-sized terrestrial or semi-arboreal species (average SVL = 712 mm; *N* = 76), with diurnal activity (Marques et al. 2017). This species occurs in the Atlantic Forest, Caatinga, Cerrado, Pantanal (Marques et al. 2005, 2015, Guedes et al. 2014, Mesquita et al. 2018). In the PEC it occurs in all states (Fig. 10E), being found in Forest, Brejos Nordestinos, Tabuleiros, and urban areas (França et al. 2012, Pereira Filho et al. 2017, Sampaio et al. 2018). *Philodryas nattereri* feeds on lizards, mammals, amphibians, snakes, and birds (Mesquita et al. 2011b). Its litter can range from 4 to 13 eggs (Vitt and Vangilder 1983, Mesquita et al. 2009).

*Philodryas olfersii* (Lichtenstein, 1823) – A moderate-sized terrestrial or semi-arboreal species (average SVL = 562 mm; *N* = 123), with diurnal activity (Marques et al. 2017). This species occurs in the Atlantic Forest, Caatinga, Cerrado, Pantanal and Pampas (Marques et al. 2005, 2015, 2019, Bérnils et al. 2007, Guedes et al. 2014). In the PEC it occurs in all states (Fig. 10E), being found in Forest, Brejos Nordestinos, Tabuleiros, Mangroves and urban areas (Pereira Filho and Montingelli 2011, França et al. 2012, Pereira Filho et al. 2017, Sampaio et al. 2018). *Philodryas olfersii* feeds on amphibians, lizards, birds and mammals (Hartmann and Marques 2005). Its litter can range from 1 to 16 eggs (Vitt and Vangilder 1983, Fowler et al. 1998, Mesquita et al. 2009).

*Philodryas patagoniensis* (Girard, 1858) – A small to moderate sized terrestrial species (average (average SVL = 436 mm; *N* = 68), with diurnal activity (Marques et al. 2019). This species occurs in the Atlantic Forest, Caatinga, Cerrado, Pantanal and Pampas (Marques et al. 2005, 2015, 2019, Bérnils et al. 2007, Guedes et al. 2014). In the PEC it occurs in the states of Pernambuco, Paraíba, and Rio Grande do Norte (Fig. 10E), being found in Forest, Tabuleiros, Restingas, and urban areas (França et al. 2012, Pereira Filho et al. 2017, Sampaio et al. 2018). *Philodryas patagoniensis* feeds on amphibians, lizards, mammals, birds, and snakes (Hartmann and Marques 2005). Its litter can range from 3 to 19 eggs (Fowler et al. 1998).

*Phimophis guerini* (Duméril, Bibron & Duméril, 1854) – A small to moderate sized terrestrial species (average SVL = 497 mm; *N* = 15), with nocturnal activity (Marques et al. 2017). This species occurs in the Atlantic Forest, Caatinga, Cerrado, Pampas and Pantanal (Lema 2003, Marques et al. 2005, 2015, Guedes et al. 2014, Mesquita et al. 2018). In the PEC it occurs in the states of Alagoas and Paraíba (Fig. 10F), being found in Forest and Tabuleiros (Rodrigues et al. 2015, Pereira Filho et al. 2017). *Phimophis guerini* feeds on lizards and mammals (Alencar et al. 2013). Its litter can range from 3 to 7 eggs (Gaiarsa et al. 2013).

*Pseudoboa nigra* (Duméril, Bibron & Duméril, 1854) – A moderate-sized terrestrial species (average SVL = 543 mm; *N* = 64), with nocturnal activity (Marques et al. 2019). This species occurs in the Atlantic Forest, Caatinga, Cerrado and Pantanal (Marques et al. 2005, 2015, 2019, Guedes et al. 2014). In the PEC it occurs in all states (Fig. 10F), being found in Forest, Brejos Nordestinos, Tabuleiros, and urban areas (Pereira Filho and Montingelli 2011, França et al. 2012, Pereira Filho et al. 2017, Mesquita et al. 2018). *Pseudoboa nigra* feeds on lizards, mammals, and snakes (Alencar et al. 2012). Its litter can range from 3 to 24 eggs (Orofino et al. 2010, Gaiarsa et al. 2013).

*Psomophis joberti* (Sauvage, 1884) – A small-sized terrestrial species (average SVL = 285 mm; *N* = 11), with diurnal activity (Marques et al. 2017). This species occurs in the Atlantic Forest, Amazon Forest, Caatinga and Cerrado (Guedes et al. 2014, Marques et al. 2015, Rodrigues et al. 2016, Mesquita et al. 2018). In the PEC it occurs only in the state of Paraíba (Fig. 10G), being found in Forest and urban areas (França et al. 2012, Pereira Filho et al. 2017). *Psomophis joberti* feeds on amphibians and lizards (Strussmann and Sazima 1993, Rodrigues et al. 2016). Its litter can have 7 eggs (Mesquita et al. 2009, 2011a).

*Sibon nebulatus* (Linnaeus, 1758) – A small-sized arboreal species (average SVL = 377 mm; *N* = 21), with nocturnal activity (Marques et al. 2019). This species occurs in the Atlantic Forest, Amazon Forest and can also be found on relict moist forests in Caatinga (Cunha and Nascimento 1993, Guedes et al. 2014, Marques et al. 2019). In the PEC it occurs in all states (Fig. 10G), being found in Forest, Tabuleiros, and urban areas (França et al. 2012, Rodrigues et al. 2015). *Sibon nebulatus* feeds on mollusks (Duellman 2005). Its litter can have 5 eggs (Boos 2001).

*Siphlophis compressus* (Daudin, 1803) – A moderate-sized arboreal and terrestrial species (average SVL = 527 mm; *N* = 13), with nocturnal activity (Marques et al. 2019). This species occurs in the Atlantic Forest and Amazon Forest (Cunha and Nascimento 1993, Marques et al. 2019). In the PEC it occurs in the states of Alagoas, Pernambuco, and Paraíba (Fig. 10G), being found in Forest and Tabuleiros (Roberto et al. 2015, Rodrigues et al. 2015, Pereira Filho et al. 2017). *Siphlophis compressus* feeds mainly on lizards, but may also feed on snakes (Martins and Oliveira 1998, Alencar et al. 2013). Its litter can range from 3 to 12 eggs (Martins and Oliveira 1998, Fraga et al. 2013, Gaiarsa et al. 2013).

*Taeniophallus affinis* (Günther, 1858) – A small-sized cryptozoic species (average SVL = 172 mm; *N* = 9), with nocturnal activity (Marques et al. 2019). This species occurs in the Atlantic Forest and Caatinga (Guedes et al. 2014, Marques et al. 2019). In the PEC it occurs in the states of Alagoas, Pernambuco, and Paraíba (Fig. 10H), being found in Forest, Brejos Nordestinos, and Tabuleiros (Rodrigues et al. 2015, Pereira Filho et al. 2017). *Taeniophallus affinis* feeds on lizards, amphibians, amphisbaenians, and mammals (Sousa and Cruz 2000, Barbo and Marques 2003, Zacariotti and Gomes 2010, Gomes 2012). Its litter can range from 5 to 7 eggs (Amaral 1978).

*Taeniophallus occipitalis* (Jan, 1863) – A small-sized cryptozoic species (average SVL = 272 mm; *N* = 63), with nocturnal activity (Marques et al. 2019). This species occurs in the Atlantic Forest, Caatinga, Cerrado and Pampas (Bérnils et al. 2007, Guedes et al. 2014, Marques et al. 2015, 2019). In the PEC it occurs in all states (Fig. 10H), being found in Forest, Brejos Nordestinos, Tabuleiros, and urban areas (Pereira Filho and Montingelli 2011, Rodrigues et al. 2015, Pereira Filho et al. 2017, França and França 2019). *Taeniophallus occipitalis* feeds on lizards, amphibians, and snakes (Balestrin and Di-Bernardo 2005, Gomes 2012). Its litter can have two eggs.

*Thamnodynastes almae* Franco & Ferreira, 2003 – A moderate-sized arboreal and terrestrial, with nocturnal activity (Franco and Ferreira 2002, Marques et al. 2019). This species occurs in the Atlantic Forest and Caatinga (Guedes et al. 2014, Marques et al. 2019). In the PEC it occurs only in Brejos Nordestinos in the state of Pernambuco (Fig. 10I) (Freitas et al. 2019a). *Thamnodynastes almae* feeds on amphibians and lizards (Marques et al. 2017a).

*Thamnodynastes hypoconia* (Cope, 1860) – A moderate-sized arboreal and terrestrial, with nocturnal activity (Marques et al. 2017a). This species occurs in the Atlantic Forest, Caatinga, Cerrado and Pampas (Bérnils et al. 2007, Guedes et al. 2014, Marques et al. 2015, 2019). In PEC it occurs only in the Parque Estadual Mata do Pau-Ferro, state of Paraíba, a Brejo Nordestino (Fig. 10I) (Pereira Filho et al. 2017). *Thamnodynastes hypoconia* feeds on amphibians and lizards (Bellini et al. 2013). Its litter can range from 4 to 13 hatchlings (Bellini et al. 2013).

*Thamnodynastes pallidus* (Linnaeus, 1758) – A small-sized arboreal and terrestrial (average SVL = 325 mm; *N* = 92), with nocturnal activity (Marques et al. 2019). This species occurs in the Atlantic Forest, Amazon Forest and Caatinga (Bailey et al. 2005, Guedes et al. 2014, Marques et al. 2019). In the PEC it occurs in the states of Alagoas, Pernambuco and Paraíba (Fig. 10I), being found in Forest and Tabuleiros (Rodrigues et al. 2015, Pereira Filho et al. 2017). *Thamnodynastes pallidus* feeds on amphibians (Guedes et al. 2014, Protázio et al. 2017). Its litter can range from 3 to 6 hatchlings (Cunha and Nascimento 1981, Araújo et al. 2018).

*Thamnodynastes phoenix* Franco, Trevine, Montingelli & Zaher, 2017 – A small to moderate size arboreal and terrestrial, with nocturnal activity (Franco et al. 2017, Marques et al. 2017a). This species occurs in the Atlantic Forest, Caatinga and Cerrado (Guedes et al. 2014, Franco et al. 2017, Freitas et al. 2019a). In the PEC it occurs only in Brejos Nordestinos of the state of Pernambuco (Fig. 10I) (Freitas et al. 2019a). *Thamnodynastes phoenix* feeds on amphibians (Pergentino and Ribeiro 2017).

**Figure 11.**
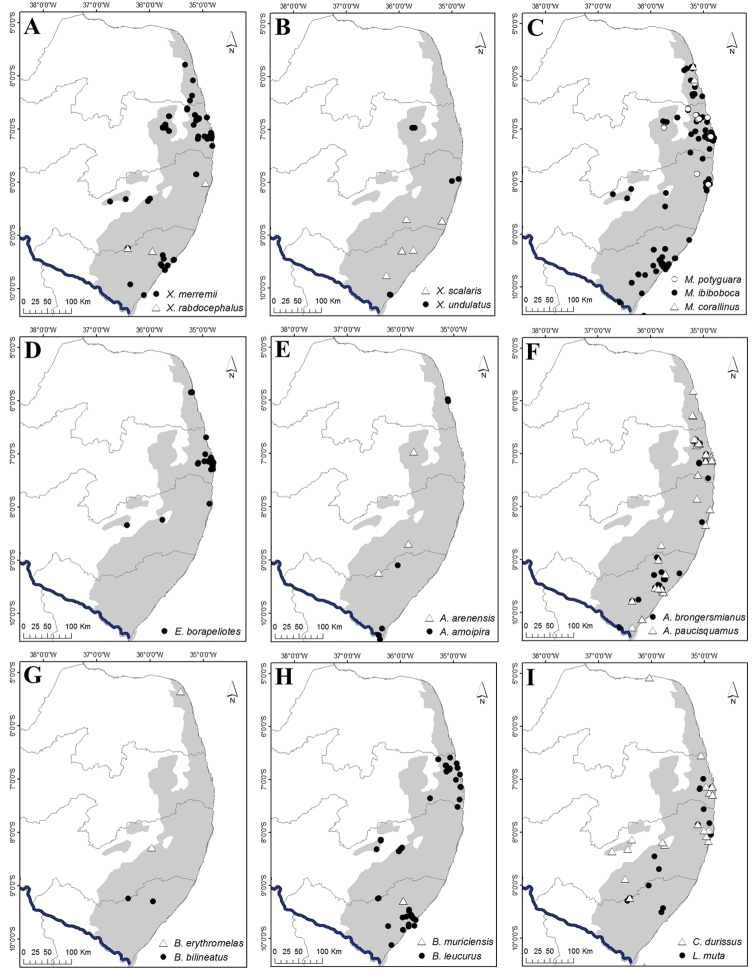
Geographic distribution records for snakes of the Pernambuco Endemism Center (PEC). **A***Xenodon merremii* and *X. rabdocephalus***B***Xenopholis scalaris* and *X. undulatus***C***Micrurus corallinus*, *M. ibiboboca* and *M. potyguara***D***Epictia borapeliotes***E***Amerotyphlops amoipira* and *A. arenensis***F***A. brongersmianus* and *A. paucisquamus***G***Bothrops bilineatus* and *B. erythromelas***H***B. leucurus* and *B. muriciensis***I***Crotalus durissus* and *Lachesis muta*.

*Xenodon merremii* (Wagler, 1824) – A small to moderate size species (average SVL = 446 mm; *N* = 97), with diurnal activity (Marques et al. 2019). This species occurs in the Atlantic Forest, Caatinga, Cerrado, Pampas Pantanal (Marques et al. 2005, 2015, 2019, Bérnils et al. 2007, Guedes et al. 2014). In the PEC it occurs in all states (Fig. 11A), being found in Forest, Brejos Nordestinos, Tabuleiros, and urban areas (Pereira Filho and Montingelli 2011, França et al. 2012, Rodrigues et al. 2015). *Xenodon merremii* feeds on amphibians (Vitt and Vangilder 1983, Mesquita et al. 2009). Its litter can range from 4 to 30 eggs (Gaiarsa et al. 2013).

*Xenodon rabdocephalus* (Wied-Neuwied, 1824) – A moderate-sized terrestrial species (average SVL = 630 mm; *N* = 2), with diurnal activity (Marques et al. 2019). This species occurs in the Atlantic Forest, Amazon Forest and Cerrado (Cunha and Nascimento 1993, Marques et al. 2015, 2019). In the PEC it occurs in the states of Alagoas and Pernambuco (Fig. 11A), being found in Forest. *Xenodon rabdocephalus* feeds on amphibians (Martins and Oliveira 1998). Its litter can range from 6 to 8 eggs (Martins and Oliveira 1998).

*Xenopholis scalaris* (Wucherer, 1861) – A small-sized terrestrial species (average SVL = 167 mm; *N* = 10), with nocturnal activity (Marques et al. 2019). This species occurs in the Atlantic Forest and Amazon Forest (Marques et al. 2015, 2019, França et al. 2019). In the PEC it occurs in the states of Alagoas and Pernambuco (Fig. 11B), being found in Forest. *Xenopholis scalaris* feeds on amphibians (Martins and Oliveira 1998, Bernarde and Abe 2010). Its litter can range from 2 to 3 eggs (Martins and Oliveira 1998).

*Xenopholis undulatus* (Jensen, 1900) – A small-sized terrestrial species (average SVL = 268 mm; *N* = 2), with nocturnal activity (Marques et al. 2019). This species occurs in the Atlantic Forest, Caatinga and Cerrado (Guedes et al. 2014, Marques et al. 2015, 2019). In the PEC it occurs in the states of Alagoas, Pernambuco, and Paraíba (Fig. 11B), being found in Forest and Brejos Nordestinos (Pereira Filho et al. 2017). *Xenopholis undulatus* feeds on amphibians (Cunha and Nascimento 1993, Kokobum and Maciel 2010). Its litter can have 3 eggs (Costa et al. 2013).

### Elapidae Boie, 1827

*Micrurus corallinus* (Merrem, 1820) – A small to moderate size cryptozoic species (average SVL = 465 mm; *N* =1), with diurnal activity (Marques et al. 2019). This species occurs in the Atlantic Forest (Marques et al. 2019). In the PEC it occurs only in the state of Rio Grande do Norte (Fig. 11C), being found in Forest. *Micrurus corallinus* feeds on amphisbaenians, lizards, snakes, and caecilians (Marques and Sazima 1997). Its litter can range from 2 to 12 eggs (Azevedo 1961, Marques 1996b).

*Micrurus ibiboboca* (Merrem, 1820) – A moderate-sized cryptozoic species (average SVL = 533 mm; *N* =391), with diurnal and nocturnal activity (Marques et al. 2017). This species occurs in the Atlantic Forest and Caatinga (Marques et al. 2017a, 2019). In the PEC it occurs in all states (Fig. 11C), being found in Forest, Brejos Nordestinos, Tabuleiros, and urban areas (Pereira Filho and Montingelli 2011, França et al. 2012, Rodrigues et al. 2015, Pereira Filho et al. 2017). *Micrurus ibiboboca* feeds on amphisbaenians, snakes, and lizards (Vitt and Vangilder 1983, Mesquita et al. 2009). We found 9 to 14 vitellogenic follicles in females.

*Micrurus potyguara* Pires, Da Silva Jr, Feitosa, Prudente, Preira-Filho & Zaher, 2014 – A moderate-sized cryptozoic species (average SVL = 523 mm; *N* = 14), with diurnal and nocturnal activity (Marques et al. 2019). *Micrurus potyguara* is endemic to the PEC, occurring in the states of Pernambuco, Paraíba, and Rio Grande do Norte (Fig. 11C), being found in Forest, Tabuleiros, and urban areas (Pires et al. 2014, Rodrigues et al. 2015, França and França 2019).

### Leptotyphlopidae Stejneger, 1891

*Epictia borapeliotes* (Vanzolini, 1996) – A small-sized fossorial species (average SVL = 111 mm; *N* = 34), with diurnal and nocturnal activity (Guedes et al. 2014). This species occurs in the Atlantic Forest and Caatinga (Guedes et al. 2014, Marques et al. 2019). In the PEC it occurs in the states of Pernambuco, Paraíba, and Rio Grande do Norte (Fig. 11D), being found in Forest, Brejos Nordestinos, and in Restingas (Pereira Filho et al. 2017, Sampaio et al. 2018, Freitas et al. 2019a). *Epictia borapeliotes* feeds on arthropods (Marques et al. 2019).

### Typhlopidae Merrem, 1890

*Amerotyphlops amoipira* (Rodrigues & Juncá, 2002) – A small-sized fossorial species (average SVL = 146 mm; *N* = 3), with nocturnal activity (Marques et al. 2017). This species occurs in the Caatinga and Atlantic Forest (Brito and Freire 2012). In the PEC it occurs in the states of Alagoas and Rio Grande do Norte (Fig. 11E), being found in Restinga (Brito and Freire 2012). *Amerotyphlops amoipira* feeds on arthropods (Marques et al. 2017a).

*Amerotyphlops arenensis* Graboski, Pereira Filho, Silva, Costa Prudente & Zaher, 2015 – A small-sized fossorial species (average SVL = 148 mm; *N* = 13). This species occurs in the Atlantic Forest and Caatinga (Graboski et al. 2015, 2019). In the PEC it occurs in the states of Alagoas, Pernambuco and Paraíba (Fig. 11E), being found in Forest and Brejos Nordestinos (Roberto et al. 2012, Graboski et al. 2015). We found 7 to 8 vitellogenic follicles in females.

*Amerotyphlops brongersmianus* (Vanzolini, 1976) – A small-sized fossorial species (average SVL = 212 mm; *N* = 120), with nocturnal activity (Marques et al. 2019). This species occurs in all Brazilian biomes (Graboski et al. 2019). In the PEC it occurs in the states of Alagoas, Pernambuco and Paraíba (Fig. 11F), being found in Forest and Tabuleiros (Pereira Filho et al. 2017, Sampaio et al. 2018). This species occurs in the Atlantic Forest (Marques et al. 2019). *Amerotyphlops brongersmianus* feeds on ant larvae (Avila et al. 2006). Its litter can range from 4 to 5 eggs (Avila et al. 2006).

*Amerotyphlops paucisquamus* (Dixon, 1979) – A small-sized fossorial species (average SVL = 133 mm; *N* =153), with nocturnal activity (Marques et al. 2019). This species is endemic to the PEC, occurring in all states (Fig. 11F), being found in Forest and Tabuleiros (Rodrigues et al. 2015, Pereira Filho et al. 2017). We found four eggs in one female and another individual laid three eggs after being collected.

### Viperidae Laurenti, 1768

*Bothrops bilineatus* (Wied-Neuwied, 1821) – A small to moderate sized arboreal species (average SVL = 495 mm; *N* = 5), with nocturnal activity (Marques et al. 2019). This species occurs in the Atlantic Forest and Amazon Forest (Bernarde et al. 2011, Marques et al. 2019). In PEC occurs only in Alagoas state (Fig. 11G), being found in Forest. *Bothrops bilineatus* feeds on mammals, amphibians, birds, snakes, and lizards (Cunha and Nascimento 1993, Martins et al. 2002, Turci et al. 2009). Its litter can range from 4 to 16 hatchlings (Dixon and Soini 1986, Campbell and Lamar 2004, Grego et al. 2012, Almeida et al. 2019).

*Bothrops erythromelas* Amaral, 1923 – A small to moderate size terrestrial species (average SVL = 445 mm; *N* = 3), with nocturnal activity (Marques et al. 2017). This species occurs in the Caatinga, but can also be found in transitional areas with the Atlantic Forest (Guedes et al. 2014). In the PEC it occurs in the states of Pernambuco and Rio Grande do Norte (Fig. 11G), being found in Forest. *Bothrops erythromelas* feeds on arthropods when juveniles, and frogs, lizards, and mammals when adults (Martins et al. 2002). Its litter can range from 2 to 21 hatchlings (Barros et al. 2014, Reis et al. 2015).

*Bothrops leucurus* Wagler, 1824 – A moderate-sized terrestrial species (average SVL = 589 mm; *N* =207), with nocturnal activity (Marques et al. 2019). This species occurs in the Atlantic Forest (Marques et al. 2019). In the PEC it occurs in the states of Alagoas, Pernambuco and Paraíba (Fig. 11H), being found in Forest, Brejos Nordestinos, Tabuleiros, mangroves, and urban areas when near forest areas (Pereira Filho and Montingelli 2011, Rodrigues et al. 2015, Pereira Filho et al. 2017, França and França 2019). *Bothrops leucurus* feeds on amphibians, lizards, snakes, birds, and mammals. Its litter can range from 5 to 7 hatchlings (Lira-da-Silva et al. 1994).

*Bothrops muriciensis* Ferrarezzi & Freire, 2001 – A moderate-sized terrestrial species (average SVL = 512 mm; *N* = 6), with nocturnal activity (Marques et al. 2019). This species occurs in the Atlantic Forest (Marques et al. 2019). This species is endemic to the PEC, occurring only in the Estação Ecológica de Murici (Fig. 11H), located in the state of Alagoas, being found in Forest. See Freitas et al. (2012) for additional information on this species. As observed in other congenerics, it probably feeds on anurans and small mammals.

*Crotalus durissus* Linnaeus, 1758 – A moderate-sized terrestrial species (average SVL = 790 mm; *N* = 13), with nocturnal activity (Marques et al. 2019). This species occurs in the Atlantic Forest, Caatinga, Cerrado, Pampas, and Pantanal (Marques et al. 2005, 2015, 2019, Bérnils et al. 2007, Guedes et al. 2014). In the PEC it occurs in the states of Alagoas, Pernambuco, and Paraíba (Fig. 11I), being found in Forest, Brejos Nordestinos, and Restingas (Lira-da-silva et al. 2009, Pereira Filho and Montingelli 2011). *Crotalus durissus* feeds on mammals (Vitt and Vangilder 1983, Strussmann and Sazima 1993, Rodrigues et al. 2016). Its litter can range from 21 to 31 hatchlings (Vitt and Vangilder 1983).

*Lachesis muta* (Linnaeus, 1766) – A large size terrestrial species (average SVL = 1217 mm; *N* = 4), with nocturnal activity (Marques et al. 2019). This species occurs in the Atlantic Forest and Amazon Forest (Cunha and Nascimento 1993, Marques et al. 2019). In the PEC it occurs in the states of Alagoas, Pernambuco and Paraíba (Fig. 11I), being found in Forest (Pereira Filho et al. 2017). *Lachesis muta* feeds on mammals (Cunha and Nascimento 1993, Martins and Oliveira 1998). Its litter can range from 1 to 18 eggs (Martins and Oliveira 1998, Souza 2007, Alves et al. 2014).

## Discussion

Our results show a broad view of PEC’s snake fauna, including distribution data, natural history, and diversity. According to Marques et al. (2019), about 142 species of snakes occur in the Brazilian Atlantic Forest, the 78 species recorded in the PEC represent 51.3% of this total, which we can consider a high richness. In addition, new species are still being discovered in this region, for example, the species *D. atlantica* (Freire et al. 2010), *M. potyguara* (Pires et al. 2014), and *A. arenensis* (Graboski et al. 2015) have been described in the last ten years and at least one new species (*Caaeteboia* sp.) is being described at the moment (Pereira Filho et al. 2017).

The mixed composition of snake species that inhabit the Atlantic Forest located north of the São Francisco River can be considered a remarkable characteristic of this fauna (Pereira Filho et al. 2017). We can highlight that the main difference between the PEC and other portions of the biome is due to the large number of species of open areas and also of wide distribution that are present in this region. The PEC shares more species with the Caatinga and the Cerrado (74.3% and 56.4% of the shared species, respectively) than with the southern and southeastern regions of the Atlantic Forest (30% of the species are shared). This may be due to the fact that the PEC presents different physiognomic features, such as patches of Tabuleiros, which are natural enclaves of savannah found even in the middle of forests and which may provide adequate conditions for the establishment of populations of species from open areas (Mesquita et al. 2018). In addition, the proximity to the Caatinga may also have favoured the penetration and establishment of these populations (Pereira Filho et al. 2017). These arguments are supported by historical factors that are based on the expansion and retraction of the boundaries of dry and open habitat ecoregions, due to climatic fluctuations over geological time, which have reached coastal areas of northeastern Brazil (Ab’Saber 1977, Pennington et al. 2006). Thus, species considered previously endemic to the Caatinga, for example, *E. borapeliotes* and *E. assisi* (Guedes et al. 2014), and species considered endemic to the Cerrado, for example *C. flavolineatus* (Nogueira et al. 2010), are also abundant in the PEC.

Most reptiles are considered habitat specialists, which means that many species can only survive in one or a few distinct environments (Martins and Molina 2008). In the PEC, the great majority of snake species were found in forest areas and 26 species were collected only in this environment. Due to the occupation of the area for agriculture and urbanization, most of the forest in the PEC was lost or reduced to small fragments, mostly smaller than ten hectares, which represent less than 2% of the original coverage of the Center (Ranta et al. 1998, Tabarelli et al. 2005). This is especially worrying because species that do not use the surrounding matrix as part of their area of use or that cannot use these environments to move between the fragments, can become extinct regionally as the populations are becoming isolated, making them unviable in the long term, due to the reduced population size (Nunney and Campbell 1993). On the other hand, some species seem to be generalists in terms of habitat and can be found in different physiognomies of the PEC and even urban areas, as is the case of *B. constrictor*, *P. olfersii*, *B. leucurus* and *O. trigeminus*.

Most snake species found in the PEC mainly use soil as substrate, as well as snakes in other regions of Brazil, such as the Caatinga (Guedes et al. 2014), Atlantic Forest (Marques et al. 2017b), Cerrado (França and Braz 2013), Pantanal (Strussmann and Sazima 1993) and Amazon (Martins and Oliveira 1998, Bernarde and Abe 2006). However, PEC also harbours a great variety of semi-arboreal and arboreal species, which is a characteristic of forest biomes, such as the Atlantic Forest and Amazon (Martins and Oliveira 1998, Argôlo 2004, Marques and Sazima 2004, Bernarde and Abe 2006).

More than half of PEC snakes feed on lizards or amphibians. These types of prey are commonly found in the snake diet, although other vertebrates like mammals, birds, and snakes are also important preys (Bernarde and Abe 2006, Hartmann et al. 2009, Mesquita et al. 2009). Some species of the PEC are generalists, as boids and snakes of the genus *Philodryas* and *Oxyrhopus*. Snakes belonging to the genera *Apostolepis*, *Dipsas*, and *Atractus* have specialized diet, feeding on snakes, mollusks and earthworms, respectively, as well as the genera *Xenodon* and *Xenopholis*, which are specialists in amphibians. (Vitt and Vangilder 1983, Laporta-Ferreira et al. 1986, Cunha and Nascimento 1993, Martins and Oliveira 1998, Mesquita et al. 2009, Bernarde and Abe 2010, Fernandes et al. 2010, Kokobum and Maciel 2010).

It is important to emphasize that the PEC presents at least seven endemic species (*A. caete*, *A. maculatus*, *B. muriciensis*, *Caaeteboia* sp., *D. atlantica*, *E. cephalomaculata*, and *M. potyguara*) of which basic information on natural history and ecology are scarce. Most of these species have a very restricted distribution, have been little recorded in nature and consequently are poorly represented in scientific collections. For example, *B. muriciensis* has only nine records and was found only in a single location (Freitas et al. 2012), the *E. cephalomaculata* has seven known records and was found only in four locations (Freitas et al. 2019b) and *Caaeteboia* sp., which has only three records and should be a new species for the region (Pereira Filho et al. 2017). Moreover, some species have confused taxonomy, such as *M. ibiboboca* and *D. neuwiedi*, being a complex of different taxa. Some of these taxa could figure as endemic species in PEC in the future. Besides the endemic species, other PEC species deserve special attention due to the absence of information on natural history and ecology, for being rare in the region and for presenting a restricted distribution in the PEC, for example, *L. trefauti*, *A. potschi*, *D. sazimai*, *D. variegata*, *E. cephalostriata*, and *A. arenensis*.

The conservation status of PEC snake species is still little known. Of the 78 species registered in the region, only 25 species have been evaluated by the IUCN (International Union for Conservation of Nature) to date. On the Brazilian list of threatened species, some PEC species are present, they are: *A. amoipira*, *A. caete*, and *B. muriciensis* as “endangered” and *A. paucisquamus* and *E. cephalomaculata* as “vulnerable” (ICMBio 2018). Given the high richness of snake species, the number of endemic species and the fragmented conditions of the region’s forests, regional conservation efforts need to be intensified, because few forests north of the São Francisco River are formally protected, and the majority are small, which means that many species in the region may be threatened with extinction (Ranta et al. 1998, Uchoa Neto and Tabarelli 2002, Tabarelli et al. 2006a).

In general, many studies still need to be developed in the PEC region, so that we can better understand the snake fauna of this region. Fauna inventories in areas that are not well sampled, population dynamics studies and distribution patterns are important for better conservation planning of PEC snake species.
